# High-throughput screening of spike variants uncovers the key residues that alter the affinity and antigenicity of SARS-CoV-2

**DOI:** 10.1038/s41421-023-00534-2

**Published:** 2023-04-11

**Authors:** Yufeng Luo, Shuo Liu, Jiguo Xue, Ye Yang, Junxuan Zhao, Ying Sun, Bolun Wang, Shenyi Yin, Juan Li, Yuchao Xia, Feixiang Ge, Jiqiao Dong, Lvze Guo, Buqing Ye, Weijin Huang, Youchun Wang, Jianzhong Jeff Xi

**Affiliations:** 1 Department of Biomedical Engineering, College of Future Technology, Peking University, Beijing, China; 2 Graduate School of Chinese Academy of Medical Sciences & Peking Union Medical College, Beijing, China; 3 Division of HIV/AIDS and Sex-transmitted Virus Vaccines, Institute for Biological Product Control, National Institutes for Food and Drug Control (NIFDC), Beijing, China; 4 Institute of Health Service and Transfusion Medicine, Beijing, China; 5 Academy for Advanced Interdisciplinary Studies, Peking University, Beijing, China; 6 GeneX Health Co. Ltd, Beijing, China; 7 College of Science, Beijing Information Science and Technology University, Beijing, China

**Keywords:** Mechanisms of disease, Bioinformatics, Glycosylation

## Abstract

Severe acute respiratory syndrome coronavirus 2 (SARS-CoV-2) infection has elicited a worldwide pandemic since late 2019. There has been ~675 million confirmed coronavirus disease 2019 (COVID-19) cases, leading to more than 6.8 million deaths as of March 1, 2023. Five SARS-CoV-2 variants of concern (VOCs) were tracked as they emerged and were subsequently characterized. However, it is still difficult to predict the next dominant variant due to the rapid evolution of its spike (S) glycoprotein, which affects the binding activity between cellular receptor angiotensin-converting enzyme 2 (ACE2) and blocks the presenting epitope from humoral monoclonal antibody (mAb) recognition. Here, we established a robust mammalian cell-surface-display platform to study the interactions of S-ACE2 and S-mAb on a large scale. A lentivirus library of S variants was generated via in silico chip synthesis followed by site-directed saturation mutagenesis, after which the enriched candidates were acquired through single-cell fluorescence sorting and analyzed by third-generation DNA sequencing technologies. The mutational landscape provides a blueprint for understanding the key residues of the S protein binding affinity to ACE2 and mAb evasion. It was found that S205F, Y453F, Q493A, Q493M, Q498H, Q498Y, N501F, and N501T showed a 3–12-fold increase in infectivity, of which Y453F, Q493A, and Q498Y exhibited at least a 10-fold resistance to mAbs REGN10933, LY-CoV555, and REGN10987, respectively. These methods for mammalian cells may assist in the precise control of SARS-CoV-2 in the future.

## Introduction

Severe acute respiratory syndrome coronavirus 2 (SARS-CoV-2) causes coronavirus disease 2019 (COVID-19), which, for the past three years, has continuously threatened public health security and global economic development^1,2^. SARS-CoV-2 owns a large positive-sense single-stranded genomic RNA (~30 kb) that encodes 16 nonstructural proteins (nsp1–nsp16), 4 structural proteins, nucleocapsid (N), envelope (E), membrane (M), spike (S), and a set of accessory proteins^3,4^.

The complete in situ structure of the SARS-CoV-2 virion has been revealed, and the virion has a crown-like appearance that is densely decorated with S glycoprotein^5,6^. Generally, the homotrimer ligand S binds to the homodimer receptor angiotensin-converting enzyme 2 (ACE2), triggering membrane fusion and viral entry^7–10^. The released viral RNA then hijacks cellular metabolism and accomplishes its life cycle through translation, replication, and self-assembly processes with a fast mutational rate^11–13^.

To date, five SARS-CoV-2 variants of concern (VOCs, referred to as Alpha, Beta, Gamma, Delta, Omicron) and eight variants of interest (VOIs, referred to as Epsilon, Zeta, Eta, Theta, Iota, Kappa, Lambda, Mu) have been designated by the World Health Organization (WHO) in terms of their superior infectivity in the population (https://www.who.int)^14,15^.

According to the updated Johns Hopkins University database, there were over 675 million diagnosed COVID-19 cases and up to 6.8 million lethal events as of 1 March 2023 (https://coronavirus.jhu.edu)^16^. Currently, despite the execution of strict anti-epidemic measures (administering vaccines, wearing masks, keeping physical distance, testing nucleic acids, etc.), infection by the highly transmissible variants with strong immune evasion was still difficult to prevent. In particular, the Delta and Omicron strains, which have successively produced outbreaks with an estimated basic reproductive number (*R*_0_) > 5, could easily become the most prevalent circulating lineages in a very short period of time^17,18^.

To date, nearly 15 million SARS-CoV-2 genomes have been submitted and shared in the Global Initiative on Sharing All Influenza Data (GISAID) database (http://www.gisaid.org), in which ~30,000 variations have been annotated (https://nmdc.cn/ncovn/)^19^. To understand the virus‒host relationship in depth, a comprehensive study is urgently needed to explore the interaction between the S protein and ACE2.

The S protein is a Class-I fusion protein and is responsible for the tropism as well as the host range of SARS-CoV-2^20,21^. The full-length S gene encodes 1273 amino acids (aa), consisting of two functionally distinct subunits: S1 (1–685 aa) and S2 (686–1273 aa), and the receptor-binding domain (RBD, 319–541 aa) in the S1 subunit directly binds to ACE2^22–24^.

Glycosylation is an important post-translational modification that greatly relies on host organelles and enzymes, and glycosylation often reflects viral virulence to some extent^25^. In brief, 22 *N*-glycans and 17 *O*-glycans have already been identified in the S protein extracted from prototype virions^26,27^. Prior valuable research has used deep mutational scanning (DMS) on the RBD in *Saccharomyces cerevisiae*, which revealed constraints on protein folding and ACE2 binding^28^, led to the design of antiviral drugs to cure clinical disease^29^, and mapped specific mutations that escape antibody recognition^30–32^. Nevertheless, the glycosyl pattern in yeast is quite different from that in human cells, and the majority of the S protein has not yet been investigated.

To mimic an authentic environment for viral infection and immune evasion, intact S protein variants were surface-displayed in mammalian cells, and the S–ACE2 interaction with or without monoclonal antibody (mAb) interference was investigated using a high-throughput paradigm. For the first time, we revealed crucial residues of the full-length S variants that influenced ACE2 binding and mAb evasion. These findings contribute to the deep understanding of the S protein and enable the regular monitoring of potential risks. Moreover, the discovered mutants may be used for the design of new therapeutics against COVID-19.

## Results

### Establishing a mammalian cell assay for surface-displaying S variants

**Fig. 1:**
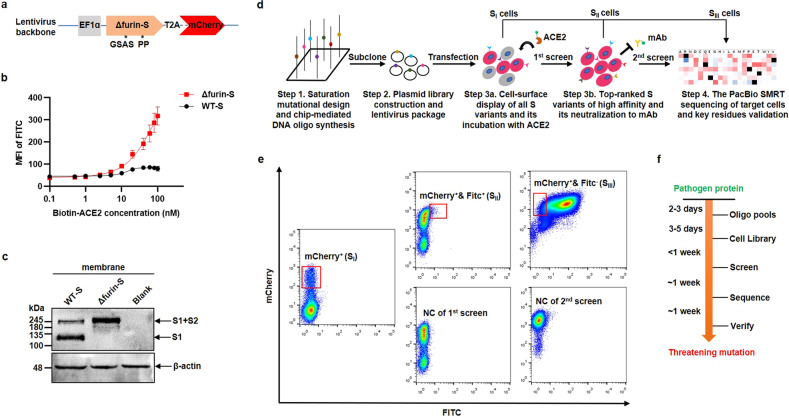
The development of robust cell surface-display platform for high-throughput screening of S variants. **a** The backbone of lentivirus plasmid, in which S variant was fused with mCherry via a T2A linker. The expression of S variant was driven by the constitutive strong EF1α promotor. **b** MFI of FITC in the WT-S and the Δfurin-S cell line, which were incubated with various concentrations of biotin-ACE2 (0, 0.1, 0.5, 1, 2.5, 5, 10, 20, 40, 60, 80, 100 nM). The non-linear regression curve (Sigmoidal, 4PL) was fitted and the bars represented SD, *n* = 2. **c** Western blotting analysis of membrane protein samples that were extracted from the WT-S, Δfurin-S, and blank HEK293T cell line, respectively. The primary antibody targeted the S1 subunit. The MW of S (S1 + S2) and S1 was migrated as ~230 kDa and ~129 kDa due to glycosylation, respectively. β-actin (~42 kDa) was used as the loading control. **d** The flowchart for high-throughput screening of S variants, from Step1 to Step 4. **e** The cell sorting graph, where the percentage of S_I_, S_II_, and S_III_ cells (red rectangle) were ensured < 10%, 1%–2%, and 5%–10%, respectively. The middle and right bottom panel represented the negative control (NC) of the 1st and 2nd screen (both showing a clear background), with no ACE2 or mAb incubation, respectively. **f** The time schedule from a new emerging pathogen protein to gain the efficient information of its threatening mutation.

The parental wild-type (WT) S protein derived from the Wuhan-Hu-1 strain (1273 aa, GenBank: QHU36824.1) was first truncated at 19 aa on the C-terminal end to facilitate protein maturation and transport (1254 aa, named WT-S)^33^. To visually trace the expression, we fused it to mCherry fluorescence with a T2A linker in a lentivirus backbone (Fig. 1a). Given that the basal level of ACE2 in human embryonic kidney 293 T cells (HEK293T) was negligible^34^, we then permanently transfected the above lentivirus particles into the cells to obtain a stable WT-S cell line. Next, the cells were incubated with varying concentrations of biotinylated human ACE2 (biotin-ACE2) and ultimately labeled with streptavidin conjugated fluorescein isothiocyanate (streptavidin-FITC). However, the mean fluorescence intensity (MFI) of FITC in the WT-S cell line remained basically unchanged regardless of ACE2 concentration (Fig. 1b, black line; Supplementary Fig. S1a, left panel). We presumed that the S protein was cleaved into S1 and S2 subunits by host furin-like proteases, thus losing binding activity to ACE2.

Therefore, we mutated WT-S by substituting “682-RRAR-685” to “GSAS” and “986-KV-987” to “PP” (named Δfurin-S), which stabilized the prefusion state as reported previously^35–37^. We then found that the MFI of FITC in the Δfurin-S cell line increased in an ACE2 dose-dependent manner (Fig. 1b, red line; Supplementary Fig. S1a, right panel). For verification, the mRNA expression of WT-S and Δfurin-S was quantified (Supplementary Fig. S1b). Moreover, western blotting analysis indicated that the WT-S protein was dynamic and partially disassociated, while the Δfurin-S protein was always integral, and was detected in both the membrane (Fig. 1c) and cytoplasm (Supplementary Fig. S1c). Similarly, the membrane of the Δfurin-S cell line emitted green fluorescence with an anti-S1 antibody (NTD-directed 4A8, RBD-directed REGN10987 and ACE2) by confocal imaging, whereas the WT-S cell line barely emitted green fluorescence (Supplementary Fig. S1d). Eventually, we constructed a lentiviral plasmid library using the Δfurin-S sequence as the template, which was more suitable for displaying the complete S protein (S1 + S2) structure on the cell surface.

### High-throughput screening of S variants for increased ACE2-binding affinity and mAb evasion

To achieve saturation mutagenesis on 1254 aa-length Δfurin-S, we preliminarily replaced each original position (except the 1^st^ Met) with the other 19 types of natural aa using the best codon (Supplementary Table S1) and synthesized ~23,807 (1253 × 19) pairs of ~100 nt single-stranded DNA oligos on two 12k chips (Fig. 1d, Step 1; Supplementary Data S1). The oligo pools were then amplified, digested, and purified as internal 45 bp primer mixtures (Supplementary Fig. S2a). After overlap PCR and Gibson assembly, a lentivirus vector carrying S variants with stochastic zero to multiple mutations was generated (Supplementary Fig. S2b). The plasmid library was extracted from all colonies on plates, and the apparent positive efficiency was determined by Sanger sequencing (Fig. 1d, Step 2 and Supplementary Fig. S2c, d). Corresponding lentivirus particles were then packaged and transfected into HEK293T cells at a low multiplicity of infection (MOI) of ~0.1, which guaranteed that a single cell received no more than one copy of the S variant and thus was convenient for further deciphering certain effective sequences.

The rationale for the selection scheme was to identify the mutation that resulted in higher binding affinity to ACE2 and stronger resistance to mAb. The stepwise process of flow cytometry was as follows. First, 10^6^–10^7^ mCherry-positive cells were harvested and cultured (mCherry^+^, Fig. 1e, left panel, named S_I_ cells), which demonstrated that the lentiviral-integrated S variants were successfully expressed in the cellular genome. To screen the variants with heightened binding affinity, total S_I_ cells were separated into two equal portions, one for RNA isolation and the other for the subsequent ACE2 incubation assay (Fig. 1d, Step 3a). Since the difference value between VOC and the Δfurin-S strain was maximized, it is easier to distinguish them at a lower concentration of ACE2 (Supplementary Fig. S1e). Hence, to deplete the S variants below the level of Δfurin-S, ~5–10 nM biotin-ACE2 was added to one portion of S_I_ cells and then they were labeled with streptavidin-FITC. The cluster of mCherry and FITC double-positive cells was collected (mCherry^+^ & FITC^+^, Fig. 1e, middle upper panel, named S_II_ cells). Moreover, to simultaneously screen the variants with higher mAb evasion, total S_II_ cells were further split into two equivalent parts, one for RNA isolation and the other for the subsequent mAb neutralization test (Fig. 1d, Step 3b). In a pilot study, commercially available mAb imdevimab (REGN10987) targeting the RBD was selected since it has been authorized for emergency use^38^. A high concentration of REGN10987 (~10 nM) was used to adequately occupy the antigen epitopes of S variants, and those that were left unbound represented mAb evaders (Supplementary Fig. S1f). To this extent, all mCherry-positive and FITC-negative cells were finally accumulated for RNA isolation (mCherry^+^ & FITC^–^, Fig. 1e, right upper panel, named S_III_ cells).

The aforementioned RNA samples of S_I_, S_II_, and S_III_ cells were extracted and reverse transcribed by a gene-specific primer (GSP) located at the 5′ terminus of mCherry (Supplementary Fig. S2e). The obtained first-strand complementary DNA (cDNA) was then amplified by unbiased high-fidelity DNA polymerase with a low cycle number (KAPA enzyme, ~20 cycles).

To directly reflect the real function of each mutational type on a holistic scale, the resulting PCR product (Supplementary Fig. S2f) was sent for third-generation DNA sequencing (PacBio SMRT Sequel-II, HiFi mode). We defined the S variants of each cluster as S_I_, S_II_, and S_III_. Generally, ~3–6 million circular consensus sequencing (CCS) reads (accuracy > 99%) were successfully output for one chip (8 million pores). As the number of CCS reads increased, the random error brought by the sequencing itself was minimized and could be recognized as a minor effect. The number of cells (the number of S variants) in each cluster was S_I_ (~10^6^–10^7^), S_II_ (~10^5^–10^6^), S_III_ (~10^5^), and we obtained CCS reads for S_I_ (~3 M), S_II_ (~3 M), and S_III_ (~0.5 M).

Moreover, only the CCS reads with the correct length (< 10 aa insertion or deletion) of S variants were eventually analyzed (Supplementary Table S3). The fold change (FC) of every mutational type was calculated as the proportion in S_II_ divided by S_I_ or the proportion in S_III_ divided by S_I_ in terms of ACE2 affinity or mAb evasion, respectively (Fig. 1d, Step 4).

This strategy to uncover a threatening mutation from a novel pathogen protein (such as the S protein of SARS-CoV-2) can be feasibly and easily operated. The entire timeline could be completed in a month or so, thus allowing enough time to respond before these harmful variants spread globally (Fig. 1f).

### An overview of the mutational effect on affinity and antigenicity

**Fig. 2:**
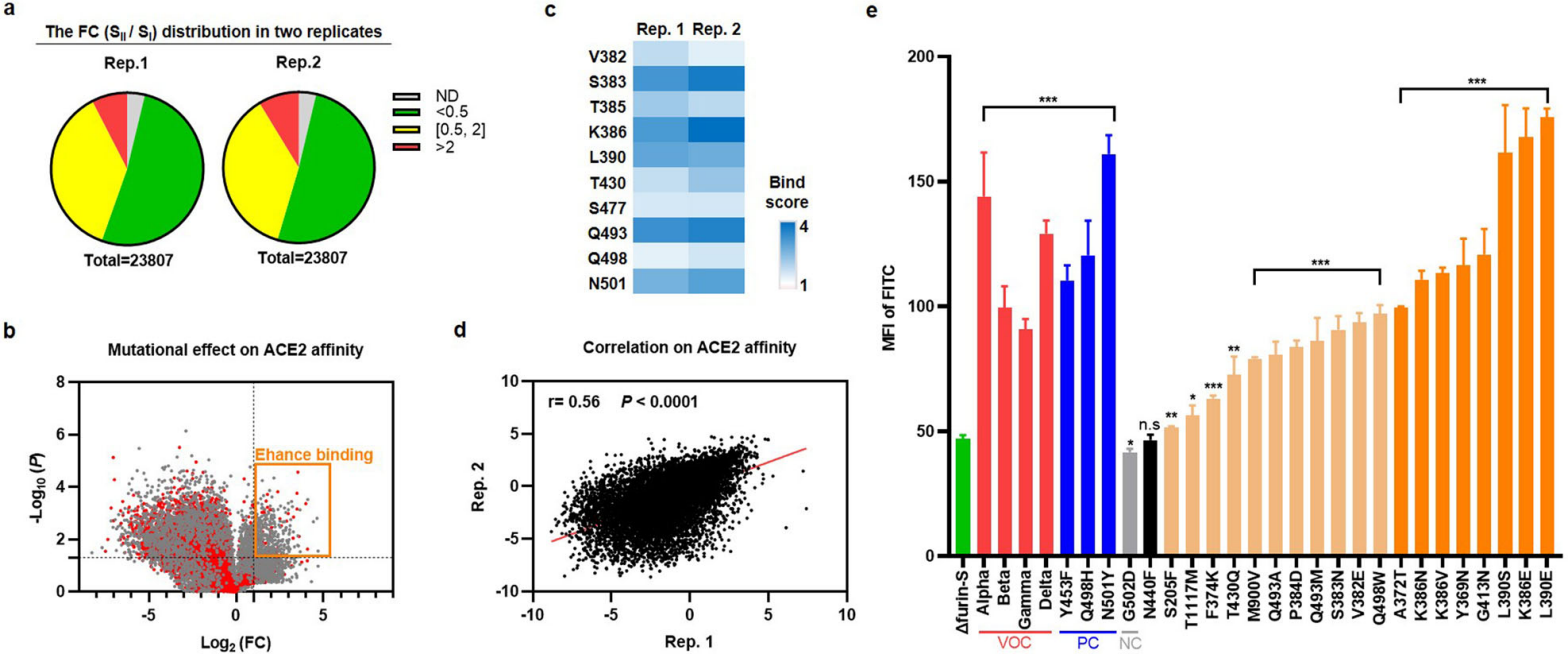
The mutational landscape and top-ranked sites validation of S variants. **a** The pie chart of the FC (S_II_ / S_I_) distribution in two replicates (named Rep.1 and Rep.2). Mutational types of ND, FC < 0.5, 0.5 ≤ FC ≤ 2, and FC > 2 were colored in gray, green, yellow, and red, respectively. **b** The volcano plot of the mutational effect on ACE2 affinity. The *x*-axis indicated the log_2_ (FC) and the *y*-axis indicated the –log_10_ (*P*). The gray and red dots represented the mutations on non-RBD and RBD region, respectively. The orange rectangle represented the “Enhance binding” mutational types that significantly heighten the affinity with ACE2, with the FC > 2 and *P* < 0.05. **c** The top ten hotspot residues on the RBD that were both enriched in two replicates, with the bind score >1. **d** The correlation analysis of log_2_ (FC) in two replicates for the ACE2 affinity, with a Pearson coefficient *r* = 0.56, *P* < 0.0001. **e** The evaluation of 20 candidates. The MFI of FITC in the VOCs (Alpha, Beta, Gamma, and Delta) was shown in red column and that in the Δfurin-S was shown in green column. Y453F, Q498H, and N501Y mutants were set as positive control (PC, blue column) while G502D mutant was set as negative control (NC, gray column). One site with no significance (n.s.) was shown in black column. Eleven tighter sites were shown in pale brown column and the eight tightest sites were shown in orange column. The *t*-test analysis was indicated as: n.s., *P* > 0.05, *P < 0.05, **P < 0.01, ***P < 0.001.

For a proof of concept, 40 non-RBD and RBD region sites with different FC values and in distinct locations were selected to evaluate ACE2-binding affinity (Supplementary Fig. S3e, f). To avoid false positives, we set the strict threshold of enrichment as FC > 2 and that of de-enrichment as FC < 0.5. For a total of 23,807 mutational types, in terms of affinity, although 3.8% were non-detected (ND), 51.3% were detrimental (FC < 0.5), 36.8% were neutral (0.5 ≤ FC ≤ 2), and 8.1% were beneficial (FC > 2) for ACE2 binding (Fig. 2a). Importantly, we observed that several well-known mutation sites in VOCs were enriched in our work (e.g., N501Y in Alpha, E484K in Beta and Gamma, L452R in Delta, N440K and S477N in Omicron). Moreover, typical K417T and K417N mutations that decrease binding affinity were not enriched (Supplementary Data S2).

The initial S_I_ cells for screening possessed an average of ~2.3 aa variations per clone, roughly following a Poisson distribution, which guaranteed that the effects of most mutations were measured in multiple genetic backgrounds (Supplementary Fig. S3a). Interestingly, we found that the average number of mutations decreased to ~1.8 aa in S_II_ cells but increased to ~2.4 aa in S_III_ cells (Supplementary Fig. S3b and Table S3). Since half of the mutational types suppressed ACE2 binding, the cells in S_I_ carrying multiple variations were inclined to lose affinity with ACE2 and were quickly selected against so that the survival cluster of S_II_ tended to possess only 0–2 mutations. However, once the binding to ACE2 was sufficient, the cells in S_II_ with more mutations provided a greater chance to block the original epitopes targeted by mAb. Thus, the final cluster of S_III_ had ≥ 3 mutations.

A volcano plot of the mutational effect on ACE2 affinity was also drawn, where the “enhance binding” region represented the significant conserved sites that emerged with an FC > 2 and *P*  < 0.05 (Fig. 2b, orange rectangle). To unveil the tolerance feature for each residue, we further calculated the bind score by averaging the FC value of all gained mutational types. Strikingly, ten key positions located on the RBD were identified: V382, S383, T385, K386, L390, T430, S477, Q493, Q498, and N501 (Fig. 2c).

Similarly, in terms of antigenicity, although 3.8% of the mutational types were ND, 42.5% were detrimental (FC < 0.5), 40.3% were neutral (0.5 ≤ FC ≤ 2), and 13.4% were beneficial (FC > 2) for mAb resistance (Supplementary Fig. S4a, b). Ten pivotal residues (S383, K386, L390, N439, N440, K444, V445, Q493, Q498, and N501) on the RBD were thought to be primarily recognized by REGN10987, including the previously reported N440, K444, and Q498 epitopes^39^ (Supplementary Fig. S4c and Data S3).

In addition, compared to the beta sheet, the bind and escape scores of the residues on the alpha helix and flexible loop were both higher in S_II_ and S_III_ cells, which suggested that the compact secondary structure was more sensitive to random mutation (Supplementary Fig. S3c, d).

The above experiments were performed in two independent biological replicates with a good correlation (Fig. 2d and Supplementary Fig. S4d). Twenty candidates with an FC > 2 were selected for the next verification. Compared with the Δfurin-S-binding affinity level, 19 sites exhibited obviously higher affinity levels (Fig. 2e and Supplementary Figs. S5a–v and S6b), of which 8 sites (Y369N, A372T, K386N, L390E, L390S, K386E, K386V, and G413N) even reached the VOC-binding affinity level (Fig. 2e, orange column).

To account for the observed changes in MFI, we first analyzed the surface expression level between these mutants. Notably, most S variants displayed very subtle differences (0.90–1.10-fold) relative to Δfurin-S (Supplementary Fig. S6a, black columns). A few exceptions (such as Delta increasing the protein yield to 1.24-fold and V382E and K386N decreasing the protein yield to 0.76-fold and 0.80-fold, respectively) were found, suggesting that other key factors (such as folding efficiency, conformational transition) may affect the affinity (Supplementary Fig. S6a, red and blue columns).

### N-glycosylation plays a critical role in affinity

Based on this phenomenon, we next attempted to uncover why a single mutation showed a prominent epistatic effect in S variants that beared multiple mutations. We investigated the structural information of the F374K, V382E, S383N, P384D, K386E, K386N, K386V, L390E, and T430Q sites (Supplementary Fig. S6c) and assumed that the nearby disulfide bonds (C379–C432 and C391–C525) might be favorably impacted so that the prefusion state would be more stable, thereby indirectly enhancing ACE2-binding affinity^40,41^.

**Fig. 3:**
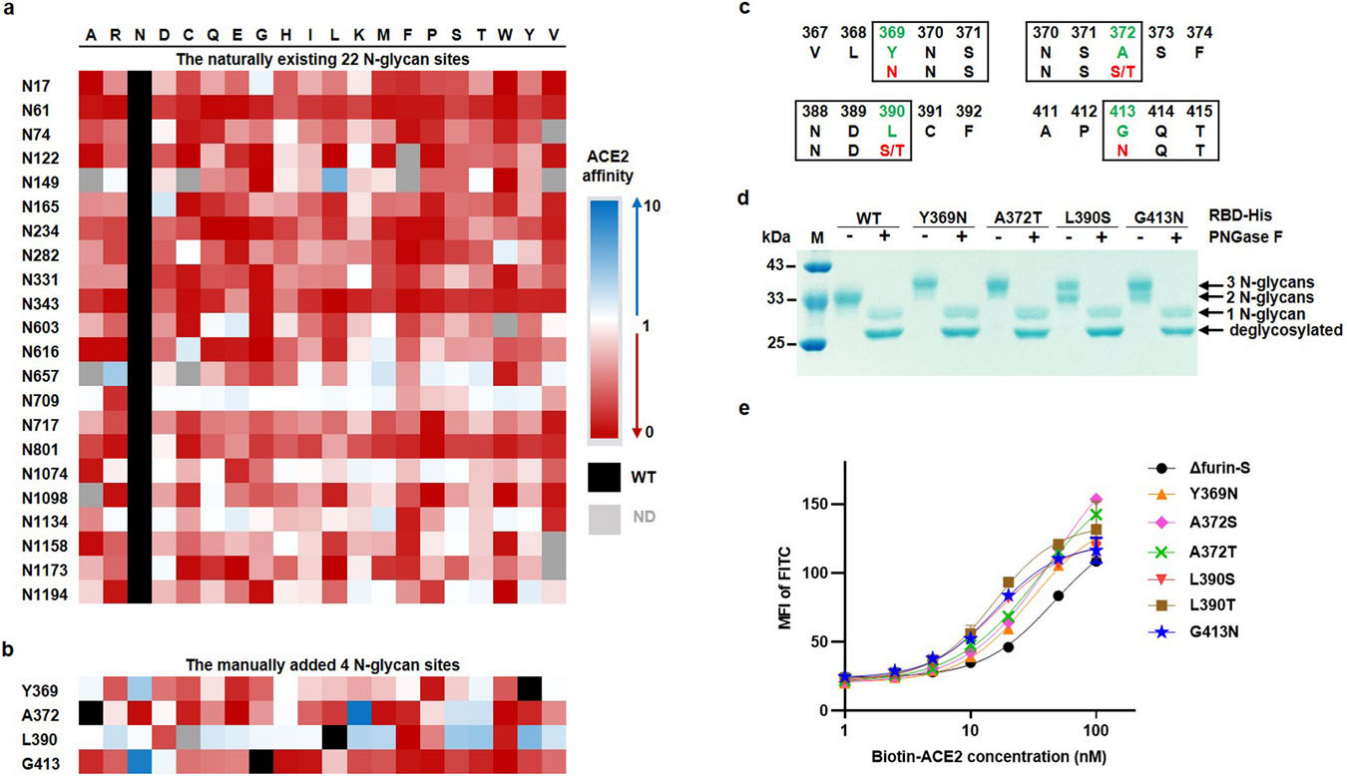
Characterization of naturally existing and manually added *N*-glycan sites. **a** The heatmap of 22 naturally existing *N*-glycan sites, in which the original asparagine (N) residues, ND mutational types were colored in black and gray, respectively. **b** The heatmap of 4 manually added *N*-glycan sites, in which the original tyrosine (Y) of 369, alanine (A) of 372, leucine (L) of 390, glycine (G) of 413 residues and ND mutational types were colored in black and gray, respectively. **c** The sequence motif of 4 manually added *N*-glycan sites (black rectangle), which was generated by the substitution of the original residue (green letter) to the novel residue (red letter). **d** The PNGase F treatment of His-tagged RBD variants. The predicted MW of three *N*-glycans, two *N*-glycans, one *N*-glycan, and zero *N*-glycan (deglycosylated) isoform was 39 kDa, 35 kDa, 31 kDa, and 27 kDa, respectively. **e** The ACE2 titration curves of Δfurin-S and six manually added *N*-glycan S variants cell lines (Y369N, A372S/T, L390S/T, and G413N).

In addition to intramolecular forces, the protruding S protein was also heavily glycosylated. An earlier study demonstrated that N-linked glycan sites profoundly contributed to viral virulence; for example, the deletion of both N331 and N343 in the RBD drastically suppressed infectivity^42^. From this point of view, we observed that the Y369N, A372S/T, L390S/T, and G413N mutants followed the canonical rules of the *N*-glycan motif (N-X-S/T, X ≠ P), which separately created the third N-linked glycosylation site in the RBD (Fig. 3c).

As a positive control in this study, we analyzed 22 naturally existing *N*-glycans^26^ by sequencing data. As expected, when these sites were ablated, the resulting variants possessed substantially decreased ACE2-binding affinity (Fig. 3a). Moreover, we tested the 4 manually added *N*-glycans by an ACE2 incubation assay. Again, all the sites exhibited the advantageous affinity over Δfurin-S (Supplementary Fig. S7a), which was consistent with the heatmap (Fig. 3b).

To verify glycosylation, His-tagged RBD carrying the four outstanding *N*-glycan mutants (Y369N, A372T, L390S and G413N) was purified by nickel beads, in which the human tPA signal peptide was added to promote its secretion. If the molecular weight (MW) was increased with the extra glycosyl chains, its migration speed would be slower, as detected by Coomassie blue-stained SDS-PAGE. In comparison with the RBD-WT with two *N*-glycans, RBD-Y369N and RBD-A372T were completely glycosylated by three *N*-glycans, while RBD-L390S and RBD-G413N were incompletely glycosylated, encompassing both three *N*-glycan and two *N*-glycan isoforms (Fig. 3d, minus sign marked lane). Moreover, we also treated the variants with glycosidase PNGase F, and all showed one *N*-glycan and zero *N*-glycan (deglycosylated) isoforms (Fig. 3d, plus sign marked lane).

Furthermore, we quantitatively measured the surface plasmon resonance (SPR) between the above monomeric RBD variants and dimeric ACE2-hFc (Supplementary Fig. S7b). Reasonably, all of these mutants displayed a mildly higher or comparable equilibrium disassociation constant (*K*D) compared to that of the WT (Supplementary Fig. S7c, d). Y369N (~13 nM) improved the affinity ~5.3-fold, mainly by decreasing the dissociation rate constant *K*_d_ (*K*_off_) ~5.6-fold. Although the association rate constant *K*_a_ (*K*_on_) was lower (~0.5-fold), A372T (~46 nM), L390S (~40 nM), and G413N (~38 nM) still elevated the affinity to 1.6–1.9-fold, which was compensated by the reduction in *K*_off_ by ~3.0-fold, ~3.2-fold, and ~3.6-fold, respectively (Supplementary Fig. S7e).

Similarly, we further calculated the apparent *K*D of each S variant using ACE2 titration curves. Compared to Δfurin-S, Y369N showed ~1.5-fold, L390S/T and G413N exhibited ~2.9–3.0-fold, while A372S/T displayed ~1.0–1.3-fold increased affinity (Fig. 3e).

### Manually adding *N*-glycans strengthens affinity but attenuates infectivity

**Fig. 4:**
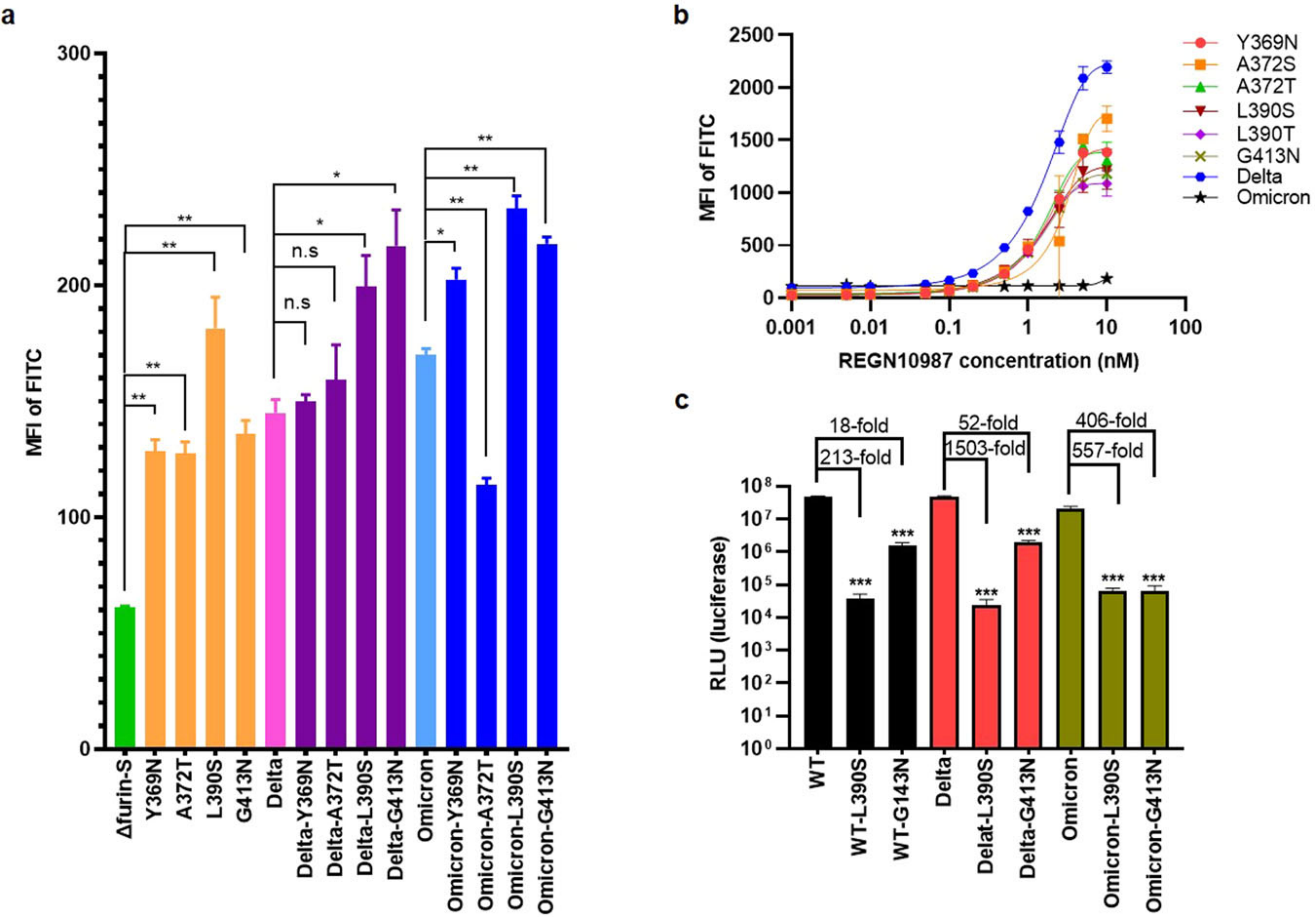
Novel adding *N*-glycan sites improved affinity but weakened infectivity. **a** MFI of FITC in the context of Δfurin-S, Delta-S, and Omicron-S, and their corresponding more *N*-glycans cell lines. The *t*-test analysis was indicated as: n.s., *P* > 0.05, *P < 0.05, **P < 0.01, ***P < 0.001. **b** MFI of FITC in manually added *N*-glycan cell lines (Y369N, A372S/T, L390S/T, and G413N), with the incubation of gradient REGN10987 (0, 0.001, 0.005, 0.01, 0.05, 0.1, 0.2, 0.5, 1, 2.5, 5, 10 nM). Delta and Omicron were set as the control. **c** The relative infectivity of WT-S, Delta-S, Omicron-S, and their corresponding L390S and G413N pseudoviruses, measuring by the read-out of luciferase signal.

Among the five VOCs, Delta and Omicron are the most threatening to the human population. To test whether heavier glycosylation would increase their infectivity, four *N*-glycan sites (Y369N, A372T, L390S, and G413N) were separately added to the S protein of Delta and Omicron variants. It is worth noting that L390S and G413N steadily improved the affinity in vitro: 1.37-fold and 1.50-fold for Delta; 1.37-fold and 1.28-fold for Omicron, respectively (Fig. 4a). In addition, these *N*-glycan mutants also exhibited stronger resistance to REGN10987 compared to that of the Delta variant, displaying the advantage of antibody escape, and Omicron was as minimally neutralized as the negative control (Fig. 4b).

We considered that the increased RBD-binding affinity of the S protein might be supported by the N-linked glycosyl chains, which expand the interface area for RBD and ACE2 binding (Supplementary Fig. S6d). Moreover, *N*-glycans may block antigen epitopes, which restrict its exposure to mAbs.

**Fig. 5:**
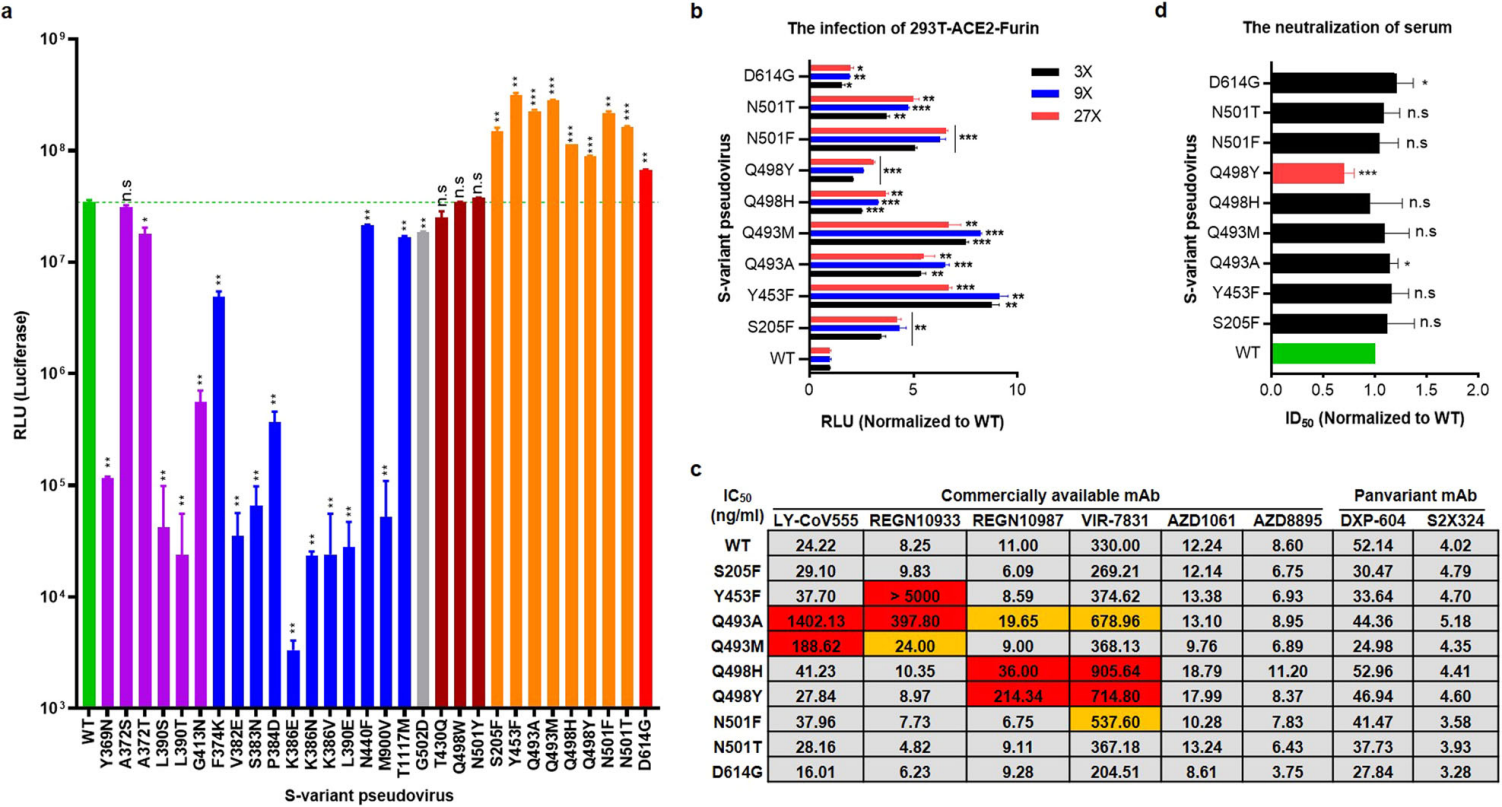
Identification of high-risk mutations that elevated the viral infectivity and immune evasion. **a** The RLU (luciferase) signal reading out from the tested S variant pseudoviruses. The green, gray, and red columns represent the WT, G502D, and D614G reference strain, respectively. The purple columns represent the *N*-glycan-related S variants (Y369N, A372S/T, L390S/T, G413N). The blue columns indicate the possible furin cleavage-related S variants (F374K, V382E, S383N, P384D, K386E, K386N, K386V, L390E, N440F, M900V, and T1117M). The brown columns represent the S variants (T430Q, Q498W, and N501Y) with comparable infectivity to WT. The orange columns indicate the S variants with improved infectivity (S205F, Y453F, Q493A, Q493M, Q498H, Q498Y, N501F, and N501T). **b** The infectivity of S variants pseudoviruses normalized to the WT (3×, 9×, and 27× dilution), which were measured in the context of 293T-ACE2-Furin cell line. D614G was set as positive control. **c** The IC_50_ (ng/mL) of high-risk S variant pseudoviruses with diverse mAbs. Each mAb was set 8 gradient dilution with two duplicates of each dilution. The red and yellow highlighted numbers show the significant and mild evasion to mAb, respectively. **d** The ID_50_ values of high-risk S variant pseudoviruses with serum antibodies. The tested sera were obtained from four female guinea pigs.

To model the entire invasion process, not only the advent of the binding stage, we recloned the 1273 aa-length S mutants without GSAS and PP mutations and further constructed a vesicular stomatitis virus (VSV)-based pseudovirus system in a biosafety level 2 laboratory following the manufacturer’s instructions^43,44^ to verify the importance of L390S and G413N. Unexpectedly, the corresponding pseudovirus greatly lost the ability to invade HEK293T cells overexpressing ACE2 and furin (293T-ACE2-Furin) in the context of WT, Delta, and Omicron variants (Fig. 4c). A similar pattern occurred in the pseudovirus with additional Y369N, A372T, or L390T mutations in the context of ancestral WT (Fig. 5a). Although the mechanism was not clear, these findings together emphasized that the *N*-glycan sites on the S variants are critically important for viral infectivity.

### Identifying potentially high-risk mutation sites

We then systematically tested 30 pseudoviruses in the context of the 293T-ACE2-Furin cell line, including the 20 preliminary candidates. However, the production of each S-variant pseudovirus was normal, and the corresponding titer was comparable (Supplementary Fig. S9a). Four *N*-glycan-related mutants (Y369N, L390S, L390T, and G413N) drastically lost infectivity (293-fold, 816-fold, 1438-fold, and 62-fold, respectively), while two mutants (A372S and A372T) on the N370 glycan site showed slightly lower (1.1-fold and 1.9-fold, respectively) infectivity (Fig. 5a, purple columns).

Nine mutational types spanning from F374 to N440 (F374K, V382E, S383N, P384D, K386E, K386N, K386V, L390E, and N440F) also decreased infectivity to different extents, varying from 10-fold to 10,000-fold (Fig. 5a, blue columns). Although they possessed a higher affinity, T430Q, Q498W, and N501Y (Fig. 5a, brown columns) basically maintained the WT infectivity level, while the non-RBD region mutations, such as M900V and T1117M (Fig. 5a, blue columns), both reduced the infectivity (661-fold and 2-fold, respectively).

Notably, eight S variants (S205F, Y453F, Q493A, Q493M, Q498H, Q498Y, N501F, and N501T) displayed even higher invasion ability than D614G, ranging from a 3–12-fold increase compared to that of WT (Fig. 5b and Supplementary Fig. S8). In addition, we also measured its infectivity in the context of three other susceptible cell lines (293T-ACE2, Huh-7, and Vero). As expected, they exhibited more aggressive invasion than that of WT, reaching or even surpassing the D614G level (Supplementary Fig. S9b–d).

However, no obvious linear positive correlation was detected between affinity and infectivity (Supplementary Fig. S9i). We proposed that these mutants may prolong the disassociation time of S1–ACE2, impair the furin-cleavage activity between S1 and S2, or change the conformation of the postfusion state, which all suppress viral entry^45–47^. Thus, we extracted the protein from the above-mentioned pseudoviruses and analyzed the composition or state of their S protein variants in vitro. Remarkably, we found that the ratio of S2/S was linearly correlated and could be used to predict infectivity (Supplementary Fig. S9j).

To support this finding, the mutants with unchanged basic properties (S205F, Y453F, Q498Y, N501F, and N501T) or more sensitive furin-cleavage activity (Q493A, Q493M, Q498H, and D614G) displayed higher infectivity than that of WT (Supplementary Fig. S9e, f); the mutants with a smaller furin-cleavage proportion (T430Q, Q498W, N501Y, and A372S) showed comparable infectivity to WT, which was possibly compensated by the high affinity (Supplementary Fig. S9f, g); the mutants with lower furin-cleavage activity (N440F, T1117M, and A372T) slightly reduced the infectivity (Supplementary Fig. S9f, g), while the other mutants with inactivated furin-cleavage capacity (M900V, Y369N, L390S/T, G413N, F374K, V382E, S383N, P384D, K386E/N/V, and L390E) largely attenuated the infectivity (Supplementary Fig. S9f–h). In addition, although the S2/S ratio of G502D was larger than that of WT, it still mildly decreased infectivity, mainly owing to its weaker binding affinity to ACE2 (Supplementary Fig. S9f). Detailed structural information is needed to explain the mechanism by which furin-cleavage activity was affected by these mutations.

Furthermore, we selected the above high-risk mutants and investigated their inhibition concentration (IC_50_) against diverse commercially available mAbs, including bamlanivimab (LY-CoV555), REGEN-COV (casirivimab (REGN10933), imdevimab (REGN10987)), sotrovimab (VIR-7831), evusheld (cilgavimab (AZD1061) and tixagevimab (AZD8895)), and two recently published panvariant antibodies, DXP-604 and S2X324^48,49^ (Supplementary Fig. S10).

Subsequently, compared with WT, Q493A and Q493M exhibited higher resistance to LY-CoV555 (Fig. 5c, 58-fold and 8-fold, respectively); Y453F and Q493A obviously prevented the neutralization of REGN10933 (Fig. 5c, 606-fold and 48-fold, respectively); Q498Y and Q498H exhibited strong evasion of REGN10987 and VIR-7831 (Fig. 5c, 19-fold and 3-fold, respectively). Moreover, Q498Y also exhibited broader defense against serum antibodies (Fig. 5d).

## Discussion

For the past three years, SARS-CoV-2 has driven a global epidemic and is thought to be one of the most contagious pathogens in human history. To cope with the ongoing challenge, diverse forms of vaccines (inactivated whole virion, LNP-encapsulated mRNA, circular RNA, RBD dimer, live-attenuated influenza-based dNS1-RBD, adenovirus-vectored Ad26-S.PP, etc.) have been employed to fight against viral infection^50–53^. Similarly, mAbs isolated from the serum of convalescent patients and humanized mice and single-domain nanobodies (Nbs) produced from the immunized camelid also act as an effective inhibitors^54–57^.

However, the genome of SARS-CoV-2 evolves rapidly, particularly the S protein, which affects the binding affinity to cognate receptor ACE2. To survive in the host, SARS-CoV-2 tends to have higher infectivity but lower mortality. In fact, patients diagnosed with a positive nucleic acid test usually exhibit mild or no clinical symptoms, making the infection difficult to trace and control. In addition, variation within the S protein seems to randomly occur in the host cell, so the next dominant strain is still a mystery.

At the present stage, Omicron and its novel sublineages (BA.2, BA.4, and BA.5) are transmitted imperceptibly, which puts a large number of individuals at risk of infection^58^. Moreover, the neutralization potency of many mAbs was immensely suppressed by Omicron^48^. In addition, zoonotic transmission from human beings to animals and, vice versa, has always been a matter of concern^59,60^. Thus, it is imperative that the molecular basis of the S–ACE2 complex be extensively investigated.

The SARS-CoV-2 virus infects the host, hijacks cellular metabolism for self-replication (bringing in novel mutations due to error-prone RNA polymerase) and is then released, triggering the humoral immune response to produce neutralizing mAbs. Several S variants may be eliminated by the mAbs, while others may survive depending on the antibody-escape mutation. In this study, we used oligo pools (1254 × 19 = 23,807, each carrying a designed mutation) to mutate the full-length S variants at specific sites. Although it is a randomized mutagenesis technique, we optimized the dosage of templates and primers, controlled the PCR temperature, amplification cycles, and other parameters, and finally generated a high-coverage lentivirus library, which contained nearly all the point mutations of the S protein sequence (> 96%). We used site-directed saturation mutagenesis to mimic the random viral mutations that occur in the host. Using this technique, harmful mutations could be identified before their emergence in the global population. Furthermore, we assessed two characteristics of these S variants: one was the binding affinity to ACE2, which represented its invasion ability (since the initial step of viral entry is ACE2–S recognition); the other was mAb resistance (in this case, REGN10987 or others were selected as an immune pressure), which reflected its immune evasion ability.

Here, we focused on the engineering of intact S variants and first identified their complete mutational profile by conducting a high-throughput screening assay in mammalian cells. We summarized that a small fraction of mutational types significantly enhanced the binding affinity to ACE2 or the resistance to REGN10987 (~8% and ~13%, respectively). Apart from the result described in yeast^28–32^, although with a good correlation (Supplementary Fig. S11a, b), we also identified many new sites that greatly impacted both ACE2 binding (Supplementary Fig. S12) and REGN10987 evasion (Supplementary Fig. S13), and their effects relied on the trimeric form of the S variant.

In the RBD region, ten hotspot residues (V382, S383, T385, K386, L390, T430, S477, Q493, Q498, and N501) improved the binding to ACE2, of which Q493A, Q493M, Q498H, Q498Y, N501F, and N501T dramatically strengthened the infectivity. Likewise, ten vital positions (S383, K386, L390, N439, N440, K444, V445, Q493, Q498, and N501) weakened the neutralization of therapeutic mAbs, of which Q498Y showed significant escape from REGN10987. For the non-RBD mutations, we tested some top-ranked candidates and identified that the vast majority of mutations were neutral or detrimental (M900V and T1117M increased the affinity but reduced the infectivity), while S205F alone increased both the affinity and infectivity. In addition, we also highlighted that four manually added *N*-linked glycan sites (Y369N, A372T, L390S/T, and G413N) and numerous mutational types spanning from F374 to N440 (F374K, V382E, S383N, P384D, K386E, K386N, K386V, L390E, and N440F) increased the affinity to some extent but decreased the virulence concurrently, which would be an ideal low-toxic antigen to stimulate the immune response.

Moreover, the top-ranked sites with high mutation frequency were recorded from 2019.12 to 2022.11 in the GISAID database (~14 million submitted genomic sequences), and it was explicit that the mutation was always closely related to the lifetime of each VOC strain (Supplementary Fig. S14a). For instance, Delta replaced Alpha, Beta, and Gamma in 2021.05 (as L18F, E484K, A570D, T716I, P681H, S982A, D1118H, and V1176F disappeared concurrently), while Omicron replaced Delta in 2021.11 (as T19R, E156G, A222V, L452R, P681R, and D950N were eliminated concurrently). Finally, various Omicron sublineages have emerged. Several Omicron sublineages have recombined with previous Delta strains; thus, elapsed mutations such as L452R are prevalent again.

In general, the possible evolution of new S variants occurs in the context of immune pressure and leads to humoral immune escape^61^. Recently, it was reported that the trend of S evolution was epistatic and convergent^62,63^. Robustly, seven out of ten key residues (R346, K444, V445, G446, N450, L452, and Q493) were enriched in our sequencing data (Supplementary Data S2 and S3). However, the currently circulating Omicron sublineages (such as BF.7, BQ.1, and XBB) carried > 30 mutations, demonstrating that the recombinational effect of multiple sites was complex, and the next variant with a survival advantage is still hard to predict. Thus, further screening of the Omicron sequence using the saturation mutagenesis method may provide insights into its mutation direction and guide efficient vaccine design.

During our manuscript preparation, a mammalian system termed “Spike Display” was published^64^, which incorporated a prefusion-stabilized S ectodomain (1–1208 aa, 6P-D614G) connected a C-terminal transmembrane domain and linker peptide. However, its conformation and epitope accessibility might be slightly affected by the additional non-S protein peptides, and the throughput was limited as one variant was assessed at a time. In addition, some new online and preprint papers described attempts to optimize S-based immunogens with increased expression and low fusogenicity^65,66^ or to directly exploit nonreplicative VSV-G pseudotyped lentiviruses to illuminate the evolution of S variants^67^. However, the mutational range (usually < 300 aa) and the number of mutated residues (only considering the sites occurring in GISAID) were restricted, and the throughput per round of screening (~10^5^ barcoded variants) was limited (Supplementary Table S4). Notably, we assessed ACE2 binding and mAb evasion in a very similar manner: S variants (~10^6^–10^7^) were analyzed in less than a month, and the sequencing data correlated well with previous work (Supplementary Fig. S11c, d).

In conclusion, we believe that this low-cost, time-efficient and multi-factor screening approach can shed light on high-risk latent viral variants and identify conserved epitopes to guide rational antibody design (Supplementary Fig. S14b). This method could also be applied for investigating the ACE2 of other species (domestic cat, cattle, sheep, etc.) or another pathogen whose entry route depends on ligand‒receptor interactions (Supplementary Fig. S14c).

## Materials and methods

### Bacteria and cell culture

*Escherichia coli* Trans1-T1 (TransGen Biotech, Cat No.: CD501) was grown at 37 °C with LB liquid medium (10 g/L tryptone, 5 g/L yeast extract, 10 g/L NaCl, pH 7.0). An extra 1.5%–2% agar was added to LB solid plates, and the working concentration of ampicillin was 100 µg/mL.

Human HEK293T/F, 293T-ACE2, 293T-ACE2-furin, Huh-7, and Vero cells were cultured at 37 °C in a humidified 5% CO_2_ incubator. Dulbecco’s Modified Eagle Medium (DMEM) (HyClone, Cat No.: SH30243.01) with 10% FBS (HyClone, Cat No.: SH30406.05, diluted at 1:10) and 1× Antibiotic-Antimycotic (Gibco, Cat No.: 15240062, diluted at 1:100) was routinely used if the medium was not specifically mentioned. The cells were washed with 1× PBS (HyClone, Cat No.: SH3025601), digested with 0.25% trypsin-EDTA (Gibco, Cat No.: 25200056), and resuspended in FACS buffer (1× PBS with 2% FBS) before sorting. The stable cell line was preserved long-term in liquid nitrogen with 90% FBS and 10% DMSO.

### Molecular cloning and plasmid construction

For the lentivirus package, pWSLV03 (Viewsolid Biotech Co. Ltd) was first cut with *Not*I (NEB, Cat No.: R3189) and *Eco*RI (NEB, Cat No.: R3101). Then the 1254 aa-length WT-S sequence was amplified from the pCMV14-3X-Flag-SARS-CoV-2 S (Addgene, Cat No.: 145780) and ligated to obtain pWSLV03-WT-S (abbreviated here as pLV-WT-S). Furthermore, we introduced “682-RRAR-685” to “682-GSAS-685” and “986-KV-987” to “986-PP-987”, generating pWSLV03-Δfurin-S (abbreviated pLV-Δfurin-S).

The S sequences of Alpha (B.1.1.7), Beta (B.1.351), Gamma (P.1), Delta (B.1.617.2), and Omicron (B.1.1.529) were codon optimized and ordered from GENEWIZ. The top-ranked S variants were obtained by site-directed mutagenesis primers using 2× Q5 Hi-fidelity mix (NEB, Cat No.: M0492).

For protein expression, pCDNA3.1(+) was first cut with *Bam*HI (NEB, Cat No.: R3136) and *Xho*I (NEB, Cat No.: R0146), and then the 223 aa-length RBD domain of S variant (Arg319 to Phe541) was amplified and flanked by an N-terminal tPA signal peptide and a C-terminal hexa-histidine tag to obtain pCDNA3.1(+)-tPA-RBD-His (abbreviated as pCD-RBD).

For pseudovirus transfection, pCDNA3.1(+) was first cut with *Bam*HI and *Xho*I, and then the full-length S variants (1273 aa, Met1 to Stop codon *1274) were amplified and inserted to acquire pCDNA3.1(+)-S (abbreviated as pCD-S).

The primers used in this study are listed in Supplementary Table S2. The sequences of all the above plasmids were determined by Sanger sequencing (Tsingke Biological Technology) and the plasmids were extracted by an endotoxin-removal kit (TIANGEN BIOTECH, Cat No.: DP120).

### Chip design, oligo pool synthesis, and library preparation

A Custom Array 12k chip possessing 12,544 pores was used to synthetize the ssDNA oligo, as described previously^68^. The length of Δfurin-S was 1254 aa, and we aimed to substitute each position (except the start codon 1^st^ Met) with the other 19 types of aa using the optimal codon for saturation mutation. Consequently, the pool required 1253 × 19 = 23,807 pair oligos, and two 12k chips needed to be covered. In this study, the designed oligo was nearly 100 nt, which was composed of a 5′ upstream adapter (26 nt, containing 18 nt M13F primer and 8 nt *Bsp*QI restriction site), an internal mutation sequence (45 nt, containing 3 nt variation in the center), and a 3′ downstream adapter (26 nt, containing 8 nt *Bsp*QI restriction site and 18 nt M13R primer).

After synthesized, the oligo pool was amplified by M13F and M13R using 2× KAPA HIFI HotStart ReadyMix (KAPABiosystems, Cat No.: KK2602) according to a two-step PCR program. The oligo pool was used first as the template for 12 cycles and then the product from the resulting 12 cycles was used as the template for another 13 cycles. As a proof-of-concept, the double-stranded product (~100 bp) was ligated to the pEASY-Blunt Zero vector (TransGen Biotech, Cat No.: CB501) and sequenced for synthetic quality. Next, it was cut by endonuclease *Bsp*QI (NEB, Cat No: R0712) at 50 °C for 1 h. Eventually, the 5′ and 3′ terminal adapters were cleaved, and the 45-bp middle mutation sequence was excised by gel electrophoresis (3% agarose, set 6 V/cm, run 3 h) and purified by a QIAquick Gel Extraction Kit (QIAGEN, Cat No.: 28706).

The appropriate amount of these 45-bp mutation sequences was added with specific forward (F) or reverse (R) primer for 20 cycles of touch-down PCR (1 ng Δfurin-S linear dsDNA as the template), respectively. Then, we mixed the above two products together and performed overlap PCR for another 10 cycles. The final PCR products with the correct length were excised, purified, and ligated to the lentivirus vector pWSLV03 using a Gibson assembly kit (NEB, Cat No.: E5510). To gain high coverage (> 100×) of S variants, we transformed them into *Escherichia coli* Trans-T1 cells and then seeded the cells into ~100 LB solid plates (10^4^–10^5^ colony forming units (CFU) per plate). The number of bacteria within the library was ~10^6^–10^7^ CFU.

### Lentivirus production and cell transfection

HEK293T cells and the production of lentiviruses were the limiting factors in this experiment. In this regard, we always prepared enough cells to cover the library size and create the lentiviruses (> 10×).

First, we seeded the lentivirus in approximately ten 10 cm culture dishes (10^7^–10^8^ cells). In this study, we used psPAX2 as the lentiviral packaging plasmid (Addgene #12260), pMD2.G as the envelope-expressing plasmid (Addgene #12259), and the pLV series single plasmid or library as the transfer plasmid. For a 10 cm culture dish, 15 µg total plasmid (1:1:1, each 5 µg), 750 µL Opti-MEM (Gibco, Cat No: 31985070) and 30 µL Lipofectamine 3000 (Invitrogen, Cat No: L3000015) were mixed first, and then 750 µL Opti-MEM and 22.5 µL P3000 were added to the reaction together (room temperature, 10–15 min).

The above mixture was then transferred to HEK293T cells (70%–90% growth confluence), and 8–10 h later, fresh medium was supplemented to reduce cell damage. Ultimately, at 48–72 h post transfection, the supernatant was harvested, concentrated by 5× Lentivirus Precipitation Solution (TransGen Biotech, Cat No.: FV101), and stored long-term at –80 °C.

Second, to ensure that only one lentivirus particle entered a cell, we selected the appropriate titer and utilized a low dose of lentivirus. In this article, 10^7^–10^8^ HEK293T cells (~three 15 cm culture dishes) were transfected at an MOI of ~0.1, and thereafter, 10^6^–10^7^ mCherry^+^ HEK293T cells were collected as the starting pool for further screening ((the number of total cells) × (the percentage of single living cells) × (the percentage of mCherry^+^ cells)). For instance, (10^8^) × (70%) × (8%) =5.6 × 10^6^.

### SDS-PAGE and western blotting analysis

The membrane samples of the WT-S and Δfurin-S cell lines were purified by a Mem-PER™ Plus kit (Thermo Fisher, Cat No.: 89842) according to the manufacturer’s recommendations. At the same time, the corresponding cytoplasm sample was acquired by cell lysis using RIPA buffer (Solarbio, Cat No.: R0010). The above two kinds of samples were mixed with 5× protein loading buffer (Coolaber, Cat No.: SL1170) and boiled at 95 °C for 5–10 min. Then, we ran SDS-PAGE (5% stacking gel at 80 V for 20 min and 8%–10% separation gel at 120 V for 1.5 h). After that, we transferred the protein bands to a PVDF membrane at 250 mA for 2 h. To investigate the expression of S variants on the membrane, a mouse anti-SARS-CoV-2 (2019-nCoV) spike RBD (Bioss, Cat No.: bsm-41516M, advisedly diluted at 1:200) targeting the S1 domain served as the primary antibody. To investigate the expression of S variants in the cytoplasm, a SARS-CoV-2 (COVID-19) spike antibody (GeneTex, Cat No.: GTX632604, advisedly diluted at 1:1000) targeting the S2 domain served as the primary antibody. In addition, an anti-β-actin mouse monoclonal antibody (Abbkine, Cat No.: ABL1010, advisedly diluted at 1:1000) was chosen as the loading control. The goat anti-mouse IgG HRP (Abbkine, Cat No.: A21010, advisedly diluted at 1:2000) was used as the secondary antibody for the above-mentioned primary antibodies. An EasySee Western blot Kit (TransGen Biotech, Cat No.: DW101) was used to detect the intensity of the target protein.

To detect the MW difference between two *N*-glycan RBD-WT and three *N*-glycan RBD variants, Coomassie Brilliant Blue Staining Reagent (Solarbio, Cat No.: P1305) was used. The His-tagged RBD protein was deglycosylated by PNGase F at 37 °C for 1 h (NEB, Cat No.: P0704), and the parallel nontreated protein was loaded as a control. ColorMixed Protein (5–245 kDa) (Solarbio, Cat No.: PR1930) and Colour Prestained Protein (10–180 kDa) (Biosharp, Cat No.: BL712A) were used as markers.

The calculated MW of the S variant, S1 subunit, and S2 subunit was 142 kDa, 77 kDa, and 65 kDa, migrating as ~230 kDa, ~129 kDa, and ~101 kDa owing to its glycosylation, respectively. Likewise, the His-tagged RBD protein was predicted to have a MW of 27 kDa and showed a 35 kDa or 39 kDa band as a result of two or three *N*-linked glycosylations.

### Confocal imaging

To determine the localization of S variants, WT-S and Δfurin-S cell lines were separately seeded onto cell slides. After 24–36 h of culture, the cells were treated with 4% PFA at room temperature for 15 min. Next, the cells were washed three times with 1× PBS, pretreated with Human TruStain FcX (Biolegend, Cat No.: 422301) at 37 °C for 5–10 min, and incubated with various antibodies (NTD-directed 4A8 (Chemstan, Cat No.: CSMA10186Mo); RBD-directed REGN10987 (Chemstan, Cat No.: CSAD00701)) or cognate receptor biotin-ACE2 (native dimeric form, Acro Biosystems, Cat No.: AC2-H82E6) at 37 °C for 30 min or 2 h, respectively.

Then, the cells were washed three times with 1× PBS and incubated with FITC-F(ab’)2 goat anti-human IgG Fcγ (Biolegend, Cat No.: 398006) or streptavidin-FITC (Biolegend, Cat No.: 405202) at 37 °C for 30 min. Thereafter, the cells were washed three times with PBST (1× PBS with 0.5% Tween 20) and stained with DAPI (1:1000) at room temperature for 10 min. Finally, the cells were treated with Antifade Mounting Medium (Beyotime, Cat No.: P0126) and photographed by a Nikon A1R confocal microscope (60×).

### Quantitative PCR

Approximately 1 μg of total RNA from the tested S variant cell line was used. PrimeScript reagent Kit with gDNA Eraser (TaKaRa, Cat No.: RR047A) was chosen to conduct reverse transcription with RT primer mix (Oligo dT primer and random 6-mers). The generated cDNA was diluted (1:10), and SuperReal PreMix Plus (TIANGEN BIOTECH, Cat No.: FP205) was selected to quantify the initial content of each template.

The primers for the amplification of GAPDH and S protein are listed in Supplementary Table S2. The Ct value of GAPDH was ~15–18 cycles. The Ct value of each S variant was normalized to GAPDH.

### Cell sorting, RNA isolation, and reverse transcription

To ensure that the cells were in a suitable state before screening, they were given a chance to recover and proliferate following each sort, which was more beneficial for the expression and display of S variants on the cell surface.

When HEK293T cells were transfected with the lentivirus library after 36–48 h, mCherry fluorescence-positive cells (S_I_) were collected. They were then seeded on the appropriate culture dish (for example, ~1 × 10^7^ cells to a 10-cm culture dish). When the S_I_ cluster cells (mCherry^+^) reached 70%–90% growth confluence (usually after 36–48 h of culture), we added a low concentration of biotin-ACE2 (5–10 nM, 37 °C, 2 h), labeled the cells with streptavidin-FITC (diluted at 1:1000, 37 °C, kept in the dark, 30 min), reserved a portion of cells for RNA isolation, and selected mCherry and FITC double fluorescence-positive cells (S_II_) by flow cytometry.

The harvested S_II_ cluster cells (mCherry^+^ & FITC^+^) were also seeded into appropriate culture dishes (for example, ~1 × 10^6^ to a 6-cm culture dish). When the S_II_ cluster cells reached 70%–90% growth confluence, they were passaged for proliferation in a larger 10-cm or 15-cm culture dish. After that, we pretreated the cells with Human TruStain FcX (5 μL/10^6^ cells, 37 °C, 5–10 min), added a high concentration of the mAb REGN10987 (~10 nM, 37 °C, 30 min), labeled the cells with FITC-F(ab’)2 goat anti-human IgG Fcγ (diluted at 1:2000, 37 °C, kept in the dark, 30 min), reserved a portion for RNA isolation, and sorted the mCherry-positive but FITC-negative cells (S_III_). The acquired S_III_ cluster cells (mCherry^+^ & FITC^-^) were seeded into the appropriate culture dish (for example, ~1 × 10^5^ to a 3.5-cm culture dish). When the S_III_ cluster cells reached 70%–90% growth confluence, total RNA was immediately extracted.

The RNA samples of S_I_, S_II_, and S_III_ cells were isolated by a HiPure Total RNA Mini Kit (Magen, Cat No.: R4111) and reverse transcribed by a SuperScript III First-Strand Synthesis System (Invitrogen, Cat No.: 18080051) using gene-specific primers (Supplementary Table S2). The cDNA was then amplified with the specific F and R primers for 20 cycles and sent to GrandOmics, Beijing.

### PacBio SMRT sequencing and data analysis

In this study, we applied the same lentivirus library to transduce HEK293T cells and generated two independent starting pools (abbreviated as Rep.1-S_I_ and Rep.2-S_I_). For each experimental group, the final PCR product originating from S_I_, S_II_, and S_III_ cells was qualified, barcoded and spread onto the sequencing chip (8 million pores). The total obtained CCS reads were output by the sequencing company with the following parameters: min-passes 3--min-rq 0.99--min-length 100 (SNP accuracy >99%). We set a threshold as “< 10 aa insert or deletion” to filter CCS reads with correct lengths, which were utilized in further analyses. According to the number of unique CCS reads, the sequencing depths of the S_I_, S_II_ and S_III_ cell clusters in the two groups Rep.1 and Rep.2 were considered saturated (2–3× coverage, Supplementary Table S3).

Then, we calculated the FC of every mutational type as the proportion in S_II_/S_I_ and S_III_/S_I_ for ACE2 affinity and mAb evasion, respectively. The value shown on the graph is the mean of two groups. Rep. 1 and Rep. 2 were highly correlated. In terms of reproducibility, for a new lentivirus library packaged in another lab, the obtained starting pools might be different. However, if we guaranteed that the number of HEK293T cells used in every step was ample and avoided undersampling, the results of screening would be similar and comparable.

### RBD purification and SPR measurement

The RBD expression plasmid pCD-RBD was transfected into HEK293F cells and cultured in flasks. The supernatants were harvested at 72–120 h post transfection and filtered with a 0.22-μm membrane. IDA-Nickel beads (Solarbio, Cat No.: M2300) were chosen to purify the His-tagged RBD (20 mM phosphate buffer, 500 mM NaCl, with an additional 20 mM imidazole for binding, 80 mM imidazole for washing, and 500 mM imidazole for elution, pH 7.4). The extracted protein (monomer, purity >95% as evaluated by SDS-PAGE) was dissolved in 1× PBS and stored at –80 °C after dialysis.

A Biacore T200 instrument (GE Healthcare), Series S Sensor Chip CM5 (Cytiva, Cat No.: 29149603), and 1× HBS-EP^+^ running buffer (Cytiva, Cat No.: BR100669, diluted at 1:10) were used for SPR. First, all paths (1, 2, 3 and 4) were activated by NHS and EDC (Cytia, Cat No.: BR100050, 1:1 mix) at a flow rate of 8 μL/min for 420 s. Next, the ligand RBD protein was diluted with 10 mM sodium acetate (Cytiva, Cat No.: BR100350, pH 4.5) and successively immobilized onto the 2, 3, and 4 paths at ~100–200 response units (RU), flow rate 8 μL/min, 60 s. Then, the chip was blocked by 1 M ethanolamine-HCl (Cytia, Cat No.: BR100050, pH 8.5) at a flow rate of 8 μL/min for 420 s. For multicycle mode, 10 mM glycine-HCl (Cytiva, Cat No.: BR100354, pH 1.5) was chosen as the regeneration condition. The mobile analyte ACE2-hFc (SGE BIOTECH, dimer, purity >95% as evaluated by SDS-PAGE) was 2-fold serial diluted in 96-well plates (0, 1.5625, 3.125, 6.25, 12.5, 25, 50, 100 nM) and injected through all paths. The procedures were set as follows: Type High performance; Contact time 300 s; Dissociation time 600 s; Flow rate 30 μL/min; Flow path 1, 2, 3, 4; Temperature 25 °C. The final recorded RU and generated sensorgrams are shown as “2-1”, “3-1”, and “4-1”, respectively.

In this article, the amount of monomeric RBD-His immobilized on the chip was comparable and strictly controlled at a low density (100–200 RU). Given that the mobile analyte was dimeric ACE2-hFc, the 2:1 (bivalent analyte) binding model would be more in line with the actual interaction occurring. Thus, the *K*D (M) value was calculated as (*K*_d_1 (1/s)/*K*_a_1 (1/Ms)). *K*_on_ and *K*_off_ were carefully estimated according to the previous literature^69^.

### The production and titration of the pseudovirus

For production of the pseudovirus, HEK293T cells were simultaneously transfected with pCD-S series plasmid (30 μg for a 75 cm^2^ flask) and VSV-G pseudotyped virus (G*ΔG-VSV) with an MOI of ~4. At 5 h post infection, cells were washed with 1× PBS with 2% FBS and supplemented with fresh medium. After 24 h, culture supernatants containing pseudovirus were harvested, filtered with 0.45-μm filters, and stored at −80 °C.

For titration of the pseudovirus, serial dilutions were first made in 96-well plates. Next, HEK293T cells overexpressing ACE2 and furin (293T-ACE2-Furin), HEK293T cells overexpressing ACE2 (293T-ACE2), Huh-7 cells, and Vero cells were seeded. After 24 h of culture in a 5% CO_2_ incubator at 37 °C, the supernatant was discarded except for 100 μL, and the same volume of luciferase substrate (Perkin Elmer, Cat No.: 6066769) was added to each well. After reaction at ambient temperature for 2 min, 150 μL of lysate was transferred to white solid 96-well plates for the final luminescence measurement.

### The mAb or serum neutralization assay mediated by pseudovirus

Briefly, the mAb (REGN10933 (Chemstan, Cat No.: CSAD00700), REGN10987 (Chemstan, Cat No.: CSAD00701), LY-CoV555 (Chemstan, Cat No.: CSAD00702), AZD8895 (Chemstan, Cat No.: CSAD00703), AZD1061 (Chemstan, Cat No.: CSAD00704), VIR-7831 (Chemstan, Cat No.: CSAD00715), DXP-604 (Chemstan, Cat No.: CSAD00842), and S2X324 (Chemstan, Cat No.: CSAD00844)) to be tested was first serially diluted (the initial highest concentration as 30-fold, 8 gradients in a 3-fold dilution manner) and added into 96-well plates in 100 μL medium. Next, 50 μL of SARS-CoV-2 S variants corresponding to pseudotyped viruses were mixed into plates at a concentration of 1 × 10^4^–2 × 10^4^ TCID_50_/mL, followed by incubation at 37 °C for 1 h. Then, 100 μL of Huh-7 cells were seeded onto the plate at a density of 2 × 10^5^ cells/mL. After 24 h of culture in a 5% CO_2_ incubator at 37 °C, chemiluminescence detection was conducted using a luminometer (Perkin Elmer, Ensight), and the 50% inhibitory dilution (EC_50_) of the antibody was calculated by the Reed-Muench method.

Additionally, the serum to be tested was obtained from female guinea pigs, which were immunized subcutaneously with 100 μg of purified S protein of the D614G reference strain with alum adjuvant once every 14 days for three inoculations, as described previously^70^.

## Supplementary information

Supplementary Information

Supplementary Data S1. Oligo list for library construction

Supplementary Data S2. Fold change for ACE2 affinity

Supplementary Data S3. Fold change for REGN10987 evasion

## Data Availability

The raw sequencing data and original analytic code have been deposited in the OMIX, China National Center for Bioinformation/Beijing Institute of Genomics, Chinese Academy of Sciences (https://ngdc.cncb.ac.cn/omix: accession No. OMIX002658).
