# Supplementary material for: High-throughput screening of spike variants uncovers the key residues that alter the affinity and antigenicity of SARS-CoV-2
Source: Cell Discov. 2023 Apr 11;9:40. doi: 10.1038/s41421-023-00534-2 (PMC10088716; doi:10.1038/s41421-023-00534-2)
Supplement: Supplementary file 1 — Supplementary Information [file 41421_2023_534_MOESM1_ESM.pdf]

Supplementary Information

High-throughput screening of spike variants uncovers the key residues that alter the affinity and antigenicity of SARS-CoV-2

Yufeng Luo[1, †], Shuo Liu[2, 3, †], Jiguo Xue[4, †], Ye Yang[1], Junxuan Zhao[1], Ying Sun[5], Bolun Wang[1], Shenyi Yin[1], Juan Li[1], Yuchao Xia[6, 7], Feixiang Ge[1], Jiqiao Dong[6], Lvze Guo[6], Buqing Ye[1], Weijin Huang[3], Youchun Wang[2, 3, *] & Jianzhong Jeff Xi[1, *]

1. Department of Biomedical Engineering, College of Future Technology, Peking University, Beijing, China
2. Graduate School of Chinese Academy of Medical Sciences & Peking Union Medical College, Beijing, China
3. Division of HIV/AIDS and Sex-transmitted Virus Vaccines, Institute for Biological Product Control, National Institutes for Food and Drug Control (NIFDC), Beijing, China
4. Institute of Health Service and Transfusion Medicine, Beijing, China
5. Academy for Advanced Interdisciplinary Studies, Peking University, Beijing, China
6. GeneX Health Co. Ltd, Beijing, China
7. College of Science, Beijing Information Science and Technology University, Beijing, China

† These authors contributed equally: Yufeng Luo, Shuo Liu, Jiguo Xue.

* Correspondence to: Youchun Wang (wangyc@nifdc.org.cn) or Jianzhong Jeff Xi (jzxi@pku.edu.cn).

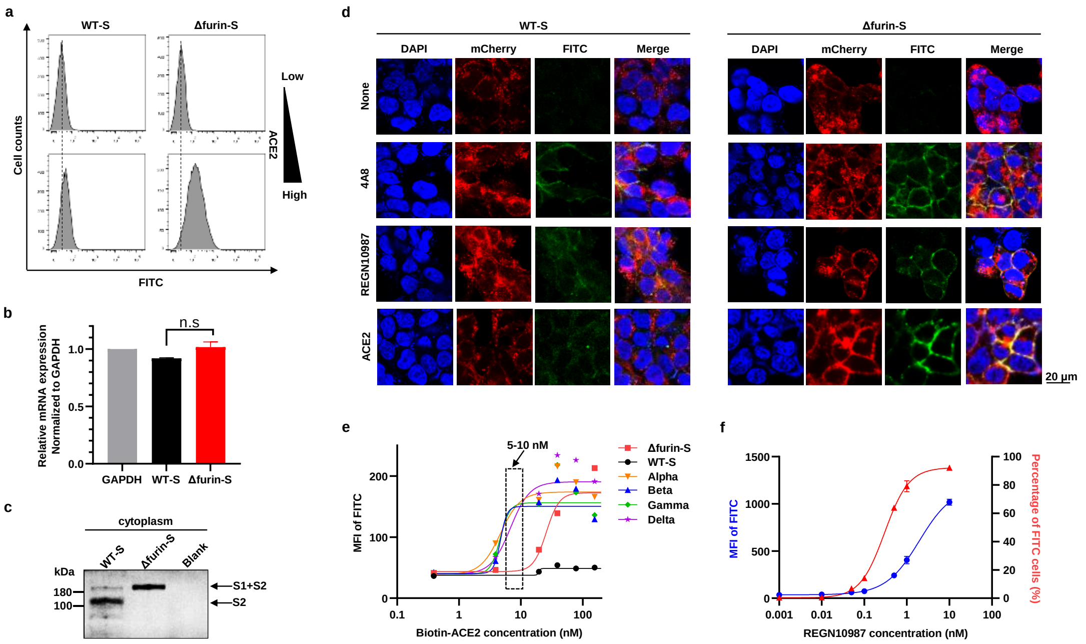

Supplementary Fig. S1:

Evaluation of the mammalian cell-surface-display system for S variants.

a, The histogram graph of WT-S and Δfurin-S cell line. The x-axis is the FITC signal and the y-axis is the cell counts. The upper panel and the bottom panel represented the incubation with low (0nM) and high (78nM) concentration of biotin-ACE2, respectively. **b,** The relative mRNA expression of S-variant in the WT-S and Δfurin-S cell line. GAPDH was served as the control gene. And n.s meant no significant difference. **c,** Western blotting analysis of cytoplasm protein samples that were extracted from the WT-S, Δfurin-S, and blank HEK293T cell lines, respectively. The primary antibody targeted the S2 subunit. The Mw of S (S1+S2) and S2 was migrated as ~230 kDa and ~101 kDa due to its glycosylation, respectively. **d,** The confocal imaging of the WT-S and the Δfurin-S cell line with the incubation of diverse antibodies (RBD-directed 4A8 and REGN10987) or cellular receptor ACE2. The microscope was photographed at 60 x. The nuclei were stained with DAPI. **e,** MFI of FITC in the WT-S, Δfurin-S, and VOC cell lines (Alpha, Beta, Gamma, Delta), incubating with gradient concentration of biotin-ACE2 (0, 0.39, 3.9, 9.75, 19.5, 39, 78, 156 nM). **f,** The percentage of FITC cells and its MFI of FITC for Δfurin-S cell line, incubating with varying concentration of REGN10987 (0, 0.001, 0.01, 0.05, 0.1, 0.5, 1, 10 nM).

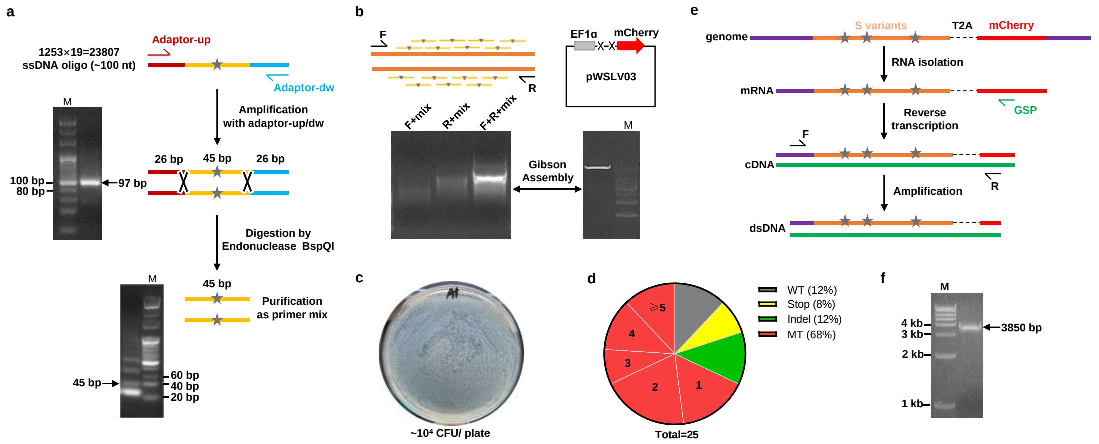

Supplementary Fig. S2:
Plasmid library construction and sequencing sample preparation.

a, The amplification, digestion, and purification of the chip-synthesized pools. 23807 pairs of ~100 nt ssDNA oligos were first amplified to dsDNA. After the digestion with BspQI (X symbol), the mutagenesis primer (45 bp) locating at the middle was cut and purified. Star mark represented the designed mutation. **b,** The ligation of the heterologous S variants fragment and the linearized lentivirus plasmid pWSLV03. The S variants were acquired by two-step overlap PCR (first F+mix 20 cycles, R+mix 20 cycles, respectively; then blended them together for another 10 cycles). The pWSLV03 was cut by NotI and EcoRI (X symbol). **c,** The bacterial transformation plate of the Gibson assembly (~10^4 CFU/plate). **d,** The mutational types were simply analysed by the Sanger sequencing. We randomly picked about 25 colonies and classified the mutational categories as follows: WT meant none mutation or synonymous mutation (n = 3), Stop meant premature stop codon (n = 2); Indel meant insertion or deletion (n = 3); MT meant correct length S variants with the number of mutations ranging from only 1 to ≥ 5 (n = 17). Thereby, the actual positive efficiency was about 70% (17/25). Theoretically, we considered that the useful counts of S variants were equal to the (total colonies) × (positive efficiency). **e,** The preparation of the transcription sample from S_{I}, S_{II}, and S_{III} cells. The first strand cDNA was obtained by GSP primer. The 5’ F and 3’ R primer was located outside the S variant, respectively. **f,** The amplification of the cDNA for third-generation sequencing (~20 cycles). The resulting PCR product was specific with a band of 3850 bp.

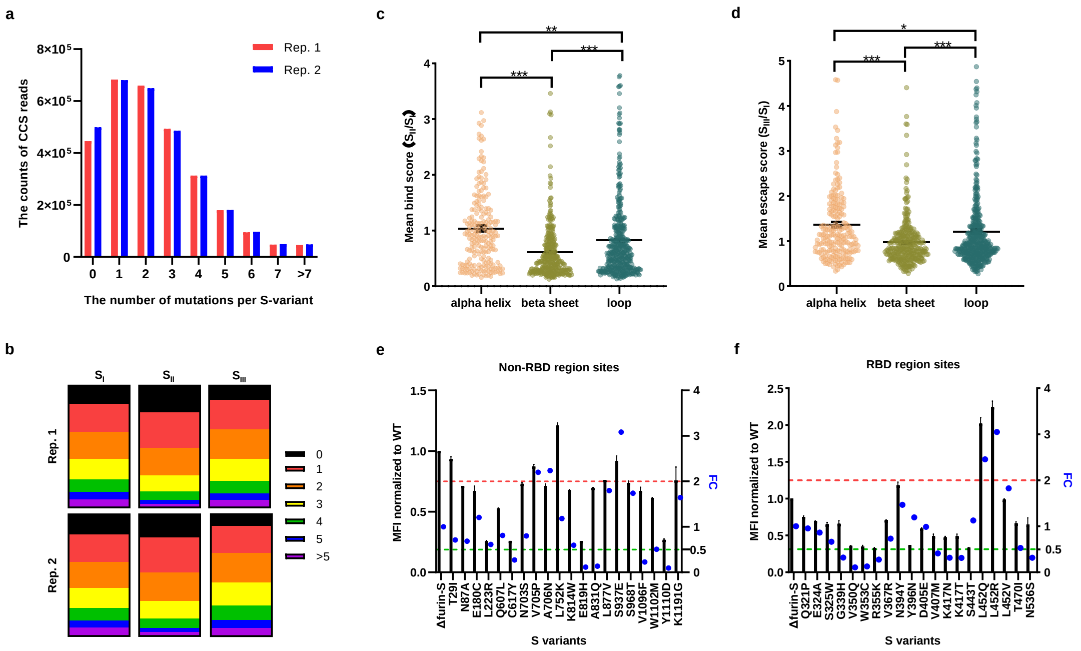

Supplementary Fig. S3:
The mutational feature of S variants during screening and verification.

a, The number of mutation per S variant and its corresponding count of CCS reads in two replicates. Rep.1 and Rep.2 were colored in red and blue column, respectively. 0 represented unmutated or those with synonymous mutations relative to Δfurin-S. **b,** The number of mutations per clone in the cluster of S_{I}, S_{II}, and S_{III}. The proportion of 0, 1, 2, 3, 4, 5, and > 5 was colored in black, red, orange, yellow, green, blue, and purple, respectively. **c-d,** The comparison of mean bind or escape scores (S_{II}/S_{I} or S_{III}/S_{I}) for residues in alpha helix, beta sheet, and loop (unstructured region). The information of secondary structure was obtained through STRIDE (http://webclu.bio.wzw.tum.de/stride/) for PDB ID: 6vyb. **e-f,** 20 mutational types in non-RBD region or RBD-region were tested for setting the suitable threshold of the enrichment and de-enrichment, respectively.

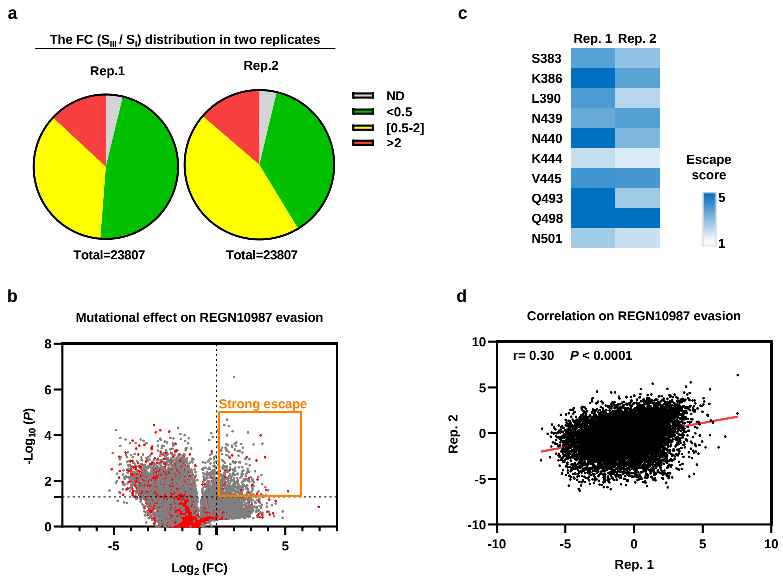

Supplementary Fig. S4:
Analyze the sequencing data for REGN10987 evasion.

a, The pie chart of the fold change (S_{III} / S_{I}) distribution in two replicates. Mutational types of ND, FC < 0.5, 0.5 ≤ FC ≤ 2, and FC > 2 were colored in gray, green, yellow, and red, respectively. **b,** The volcano plot of the mutational effect on REGN10987 evasion. The x-axis indicated the $\log_2$ (FC) and the y-axis indicated the $-\log_{10}$ (*P*). The gray and red dots represented the mutations on non-RBD or RBD region, respectively. The orange rectangle represented the "Strong escape" mutational types that significantly evaded the REGN10987, with FC > 2 and *P* < 0.05. **c,** The top ten hotspot residues (S383, K386, L390, N439, N440, K444, V445, Q493, Q498, and N501) on the RBD that were both enriched in two replicates, with the escape score > 1.5. **d,** The correlation analysis of two replicates for the REGN10987 evasion, with a Pearson coefficient r=0.30, *P* < 0.0001.

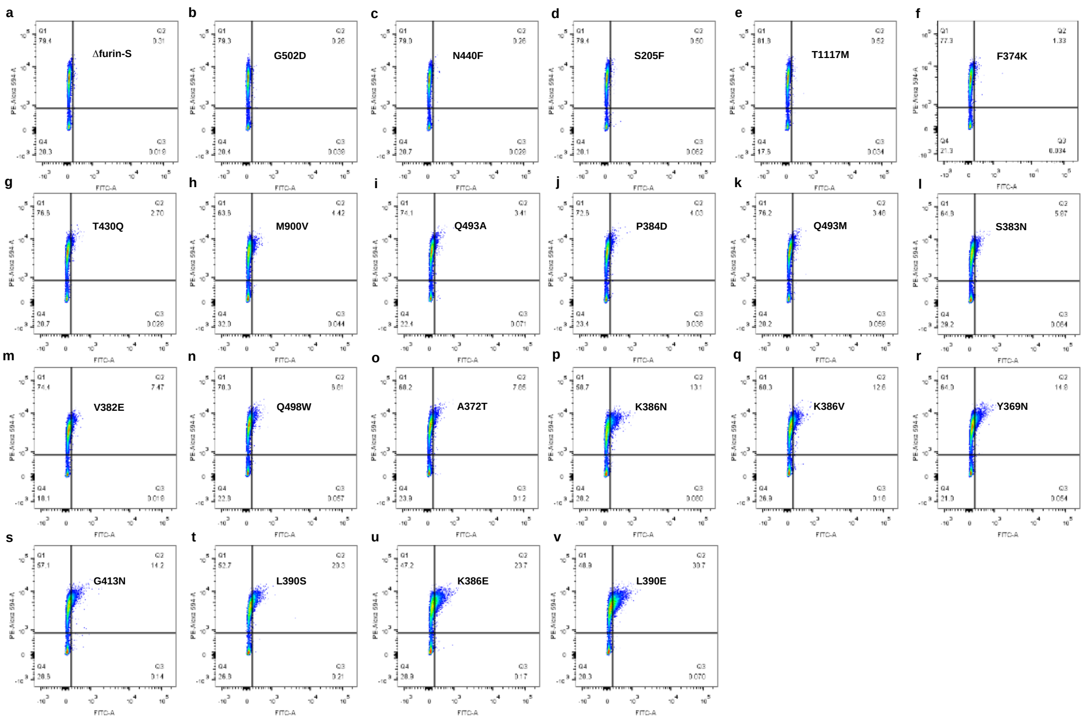

Supplementary Fig. S5:
MFI of FITC for each S variant cell lines.

a, Δfurin-S was the reference strain. **b,** G502D was set as the negative control. **c-v,** 20 candidates were tested for the validation of screening data. Each S variant was lentivially-integrated into the HEK293T cells (MOI~0.1) first and then collected for the mCherry$^+$ cells in order to ensure that the expression was stable. After incubating with biotin-ACE2 (5-10nM) and streptavidin-FITC, the sorting gate was drawn as follows: FSC-A/SSC-A for living cells; FSC-A/FSC-W and SSC-A/SSC-W for single cells; PE- Alexa 594-A/FITC-A for dividing the cluster. The final recorded events were about 20000 per sample. And the MFI of FITC was measured as (The fluorescence intensity in Q1 and Q2/ The cell counts in Q1 and Q2).

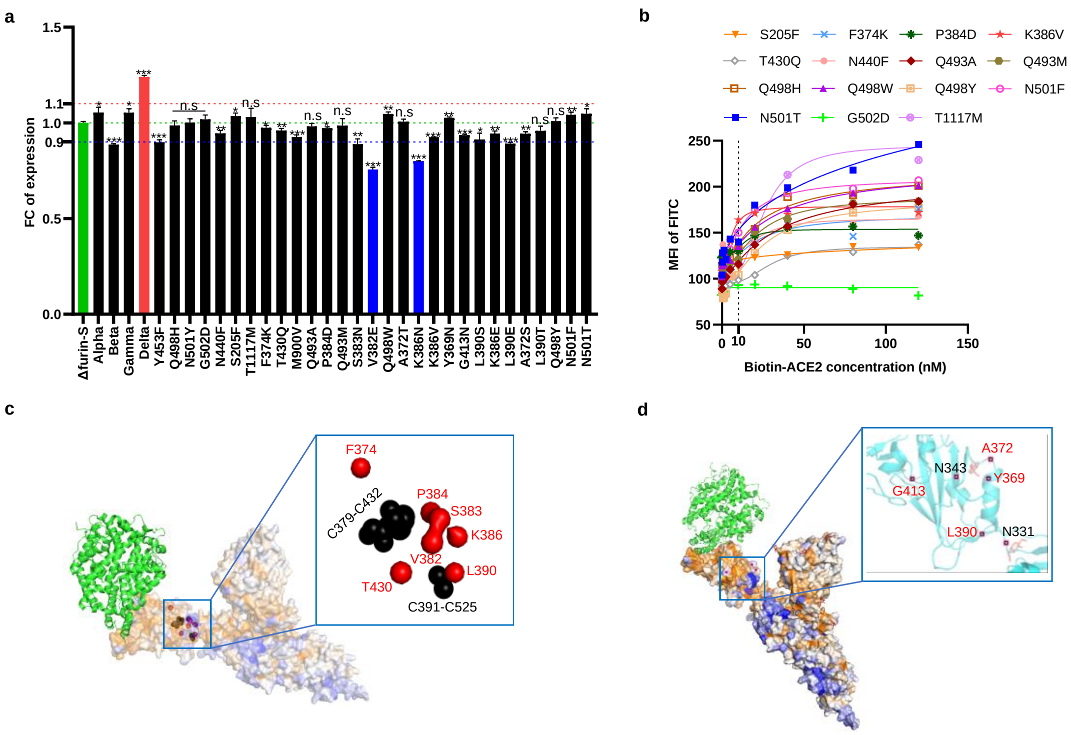

Supplementary Fig. S6:
The expression level and structural information of the tested mutants.

a, The FC of expression for each S variant, which was normalized to Δfurin-S level (green column). The obvious difference was set as > 1.1 or < 0.9. The t-test analysis was indicated as: $P > 0.05$ (n.s), $P < 0.05$ (*), $P < 0.01$ (**), $P < 0.001$ (***). **b,** Some candidates were further tested by ACE2 titration curves, which was measured with the gradient concentration (0, 0.125, 0.25, 0.625, 1.25, 2.5, 5, 10, 20, 40, 80, 120 nM). **c,** The position of the important disulfide bonds. The green cartoon structure represented the ACE2 and the surface structure indicated one S protomer (PDB ID: 7DF4). The S was pre-colored with the bind score of each residue (the bluer the tighter affinity). Two disulfide bonds (C391-C525 and C379-C432) were colored in black and the surronded residues (F374, V382, S383, P384, K386, L390, and T430) were colored in red. **d,** The position of the naturally existed N-glycans (N331 and N343) and the manually introduced N-glycans (Y369, A372, L390, and G413).

a b d

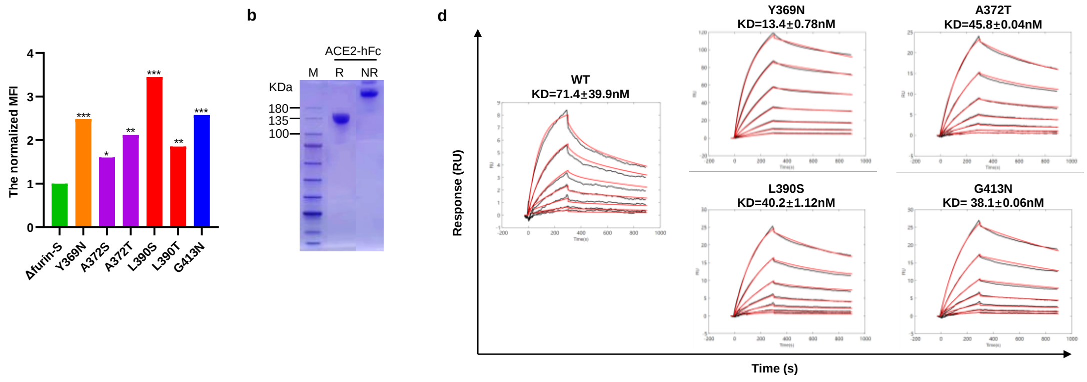

c

Sample	k_a1 (1/ Ms)	k_d1 (1/ s)	KD (M)	Immobilize (RU)	R_{max} (RU)	Chi² (RU²)	ACE2-hFc (nM)	Model
WT	4.696E+4	2.028E-3	4.319E-8	100	9.0	0.02	0	2:1 bivalent analyte
	3.371E+4	3.359E-3	9.964E-8		11.3	0.01	1.5625	
Y369N	3.549E+4	4.955E-4	1.396E-8	180	161.2	0.67	3.125	
	3.673E+4	4.718E-4	1.285E-8		154.0	0.90	6.25	
A372T	2.062E+4	9.432E-4	4.574E-8	143	37.6	0.05	12.5	
	1.870E+4	8.564E-4	4.580E-8		39.4	0.11	25	
L390S	2.226E+4	8.775E-4	3.942E-8	130	37.3	0.08	50	
	1.997E+4	8.190E-4	4.101E-8		40.9	0.09	100	
G413N	1.979E+4	7.552E-4	3.816E-8	126	44.3	0.11		
	1.930E+4	7.349E-4	3.808E-8		43.8	0.11		

e

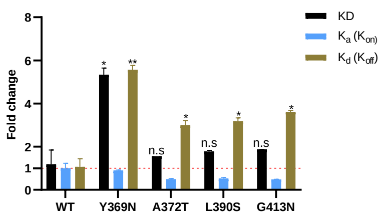

Supplementary Fig. S7:
The affinity of RBD with manually added N-glycan sites.

a, The normalized MFI of FITC in N-glycan related S variant cell lines. The t-test analysis was indicated as: $P > 0.05$ (n.s), $P < 0.05$ (*), $P < 0.01$ (**), $P < 0.001$ (***). **b,** The validation of mobile analyte ACE2-hFc, which was existed in a dimeric form (Mw of NR / R) and with a purity > 95% detecting by SDS-PAGE. **c,** SPR measurement of RBD variants (WT, Y369N, A372T, L390S, and G413N) with ACE2-hFc. Each RBD variant was first immobilized to the CM5 chip (100-200RU) by amine coupling, and then 2-fold serial dilution ACE2-hFc ranging from 0 to 100nM (0, 1.5625, 3.125, 6.25, 12.5, 25, 50, 100nM) was injected to start reaction (5min association, 10min disassociation). **d,** The sensorgram of each RBD variant, the raw data was shown in black curve and the fitted 2:1 (bivalent analyte) binding model was shown in red curve. The x-axis was the reaction time (s) and the y-axis was the response (RU). The final KD (M) was estimated by [k_d1 (1/s) / k_a1 (1/Ms)] and shown as mean ± SD, n=2. **e,** The KD, K_a (K_{on}), and K_d (K_{off}) analysis of the WT and each RBD variant. The fold change was normalized to the WT level, by calculating KD WT/ KD, K_{on}/ K_{on} WT, and K_{off} WT/ K_{off}, respectively.

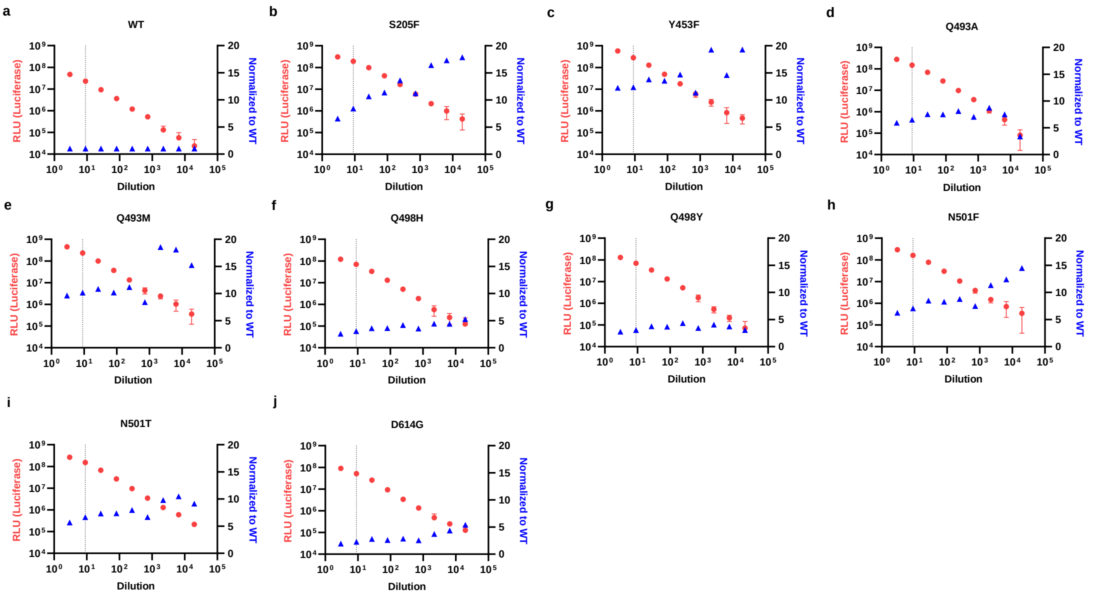

Supplementary Fig. S8:
The titration of high-risk S variants pseudoviruses.

a-j, The infectivity of serial dilution pseudovirus (3X, 9X, 27X, 81X, 243X, 729X, 2187X, 6561X, and 19683X) for WT, S205F, Y453F, Q493A, Q493M, Q498H, Q498Y, N501F, N501T, and D614G, respectively. The left y-axis represented RLU (luciferase) and the right y-axis meant the normalize to WT. Generally, 9X dilution was considered as the unified infection standard (the vertical dashed line).

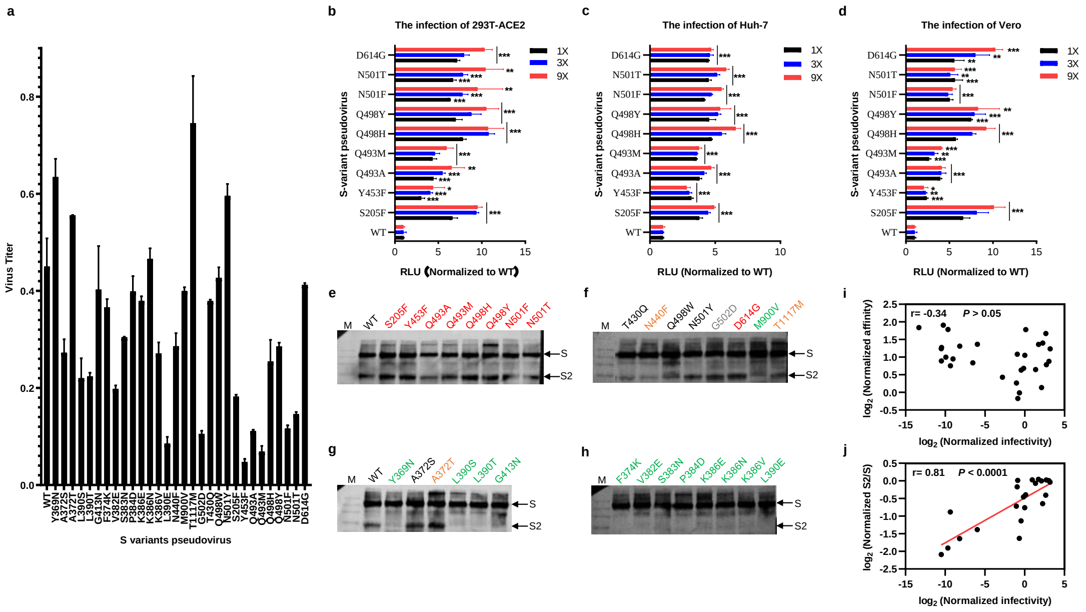

Supplementary Fig. S9:

Furin-cleavage activity affect the infectivity of S variants pseudoviruses.

a, The virus titer of the tested S variants pseudoviruses. The virus RNA was isolated and immediately quantified by the RT-PCR of VSV-P gene. **b-d,** The normalized infectivity of S variants pseudoviruses to WT (1X, 3X, and 9X dilution), which was measured in the context of 293T-ACE2, Huh-7, and Vero cell lines, respectively. D614G was set as the positive control. **e-h,** The S2/S ratio of all the tested S variants pseudoviruses. The intensity of each protein band was measured by Image J software. **i,** The correlation between infectivity (RLU normalized to WT) and affinity (MFI of FITC normalized to WT). **j,** The correlation between infectivity (RLU normalized to WT) and furin-cleavage activity (S2/S ratio normalized to WT).

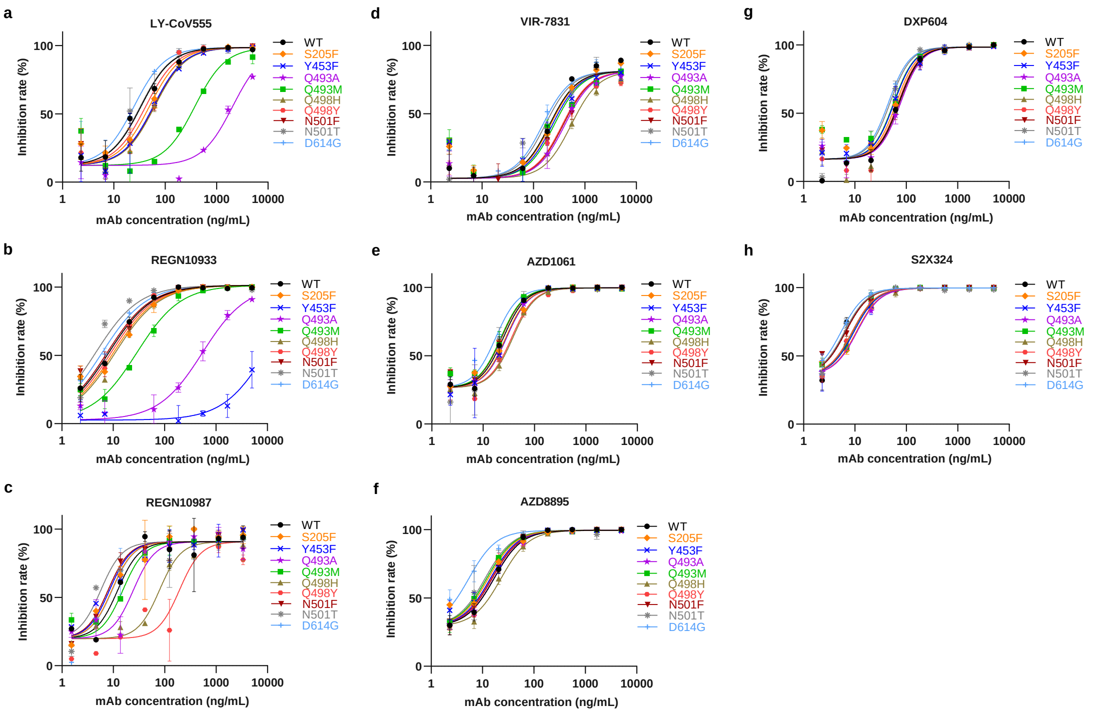

Supplementary Fig. S10:
The neutralization of high-risk S variants pseudoviruses with diverse mAbs.

a, The inhibition curves of the LY-CoV555, of which Q493A and Q493M showed significant evasion. **b,** The inhibition curves of the REGN10933, of which Y453F and Q493A showed significant evasion. **c,** The inhibition curves of the REGN10987, of which Q498Y and Q498H showed significant evasion. **d,** The inhibition curves of the VIR-7831, of which Q498H and Q498Y showed significant evasion. **e-h,** The inhibition curves of the AZD1061, AZD8895, DXP604, and S2X324, respectively. The tested high-risk S variants displayed no significant evasion.

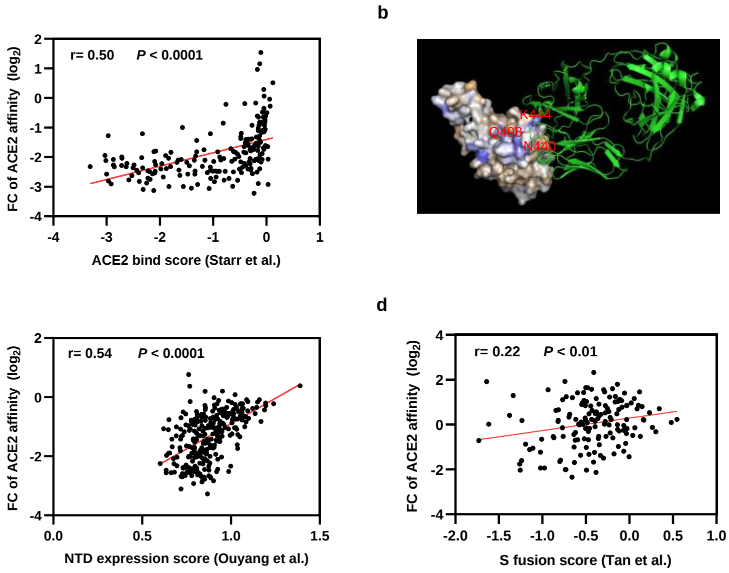

Supplementary Fig. S11:
The correlate analysis with prior published work.

a, The sequencing data comparision between our work and Starr et al.[28], in terms of ACE2 affinity. **b,** The epitope recognised by REGN10987. The green cartoon structure was REGN10987 and the surface structure was RBD, which was pre-colored with the escape score of each residue (the bluer the higher evasion). PDB ID: 6XDG. **c,** The sequencing data comparision between our work and Ouyang et al.[65], in terms of NTD expression. **d,** The sequencing data comparision between our work and Tan et al.[66], in terms of S fusogenicity.

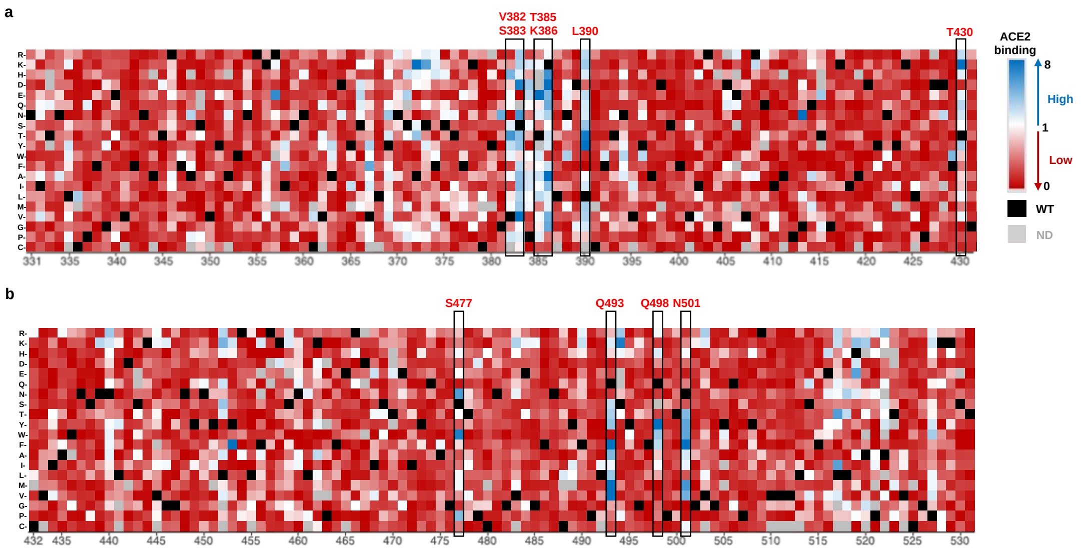

Supplementary Fig. S12:

Identify the key residues in RBD that bind with ACE2.

a, The heatmap spanning from 331-431aa, where the hotspot residues (V382, S383, T385, K386, L390, and T430) were uniquely enriched in our data. **b,** The heatmap spanning from 432-531aa, where the hotspot residues (S477, Q493, Q498, and N501) were commonly enriched in previous work[28]. The black and gray block represented the original residue (WT) and the nondetected (ND) mutational types, respectively. The red meant lower affinity and the blue meant higher affinity, respectively.

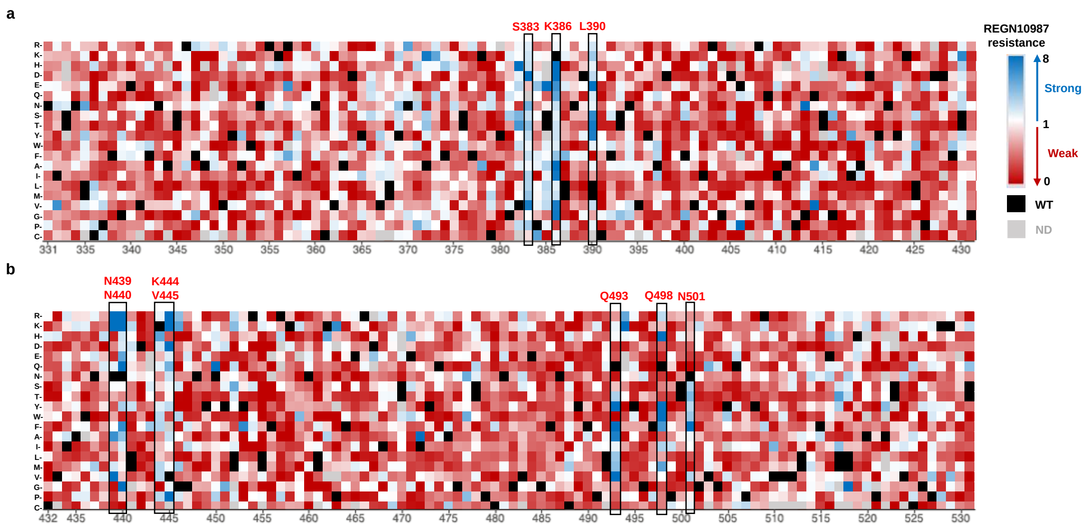

Supplementary Fig. S13:

Identify the key residues in RBD that escape from REGN10987.

a, The heatmap spanning from 331-431aa, where the hotspot residues (S383, K386, and L390) were enriched. **b,** The heatmap spanning from 432-531aa, where the hotspot residues (N439, N440, K444, V445, Q493, Q498, and N501) were enriched. The black and gray block represented the original residue (WT) and the ND mutational types, respectively. The red meant weak evasion and the blue meant strong evasion, respectively.

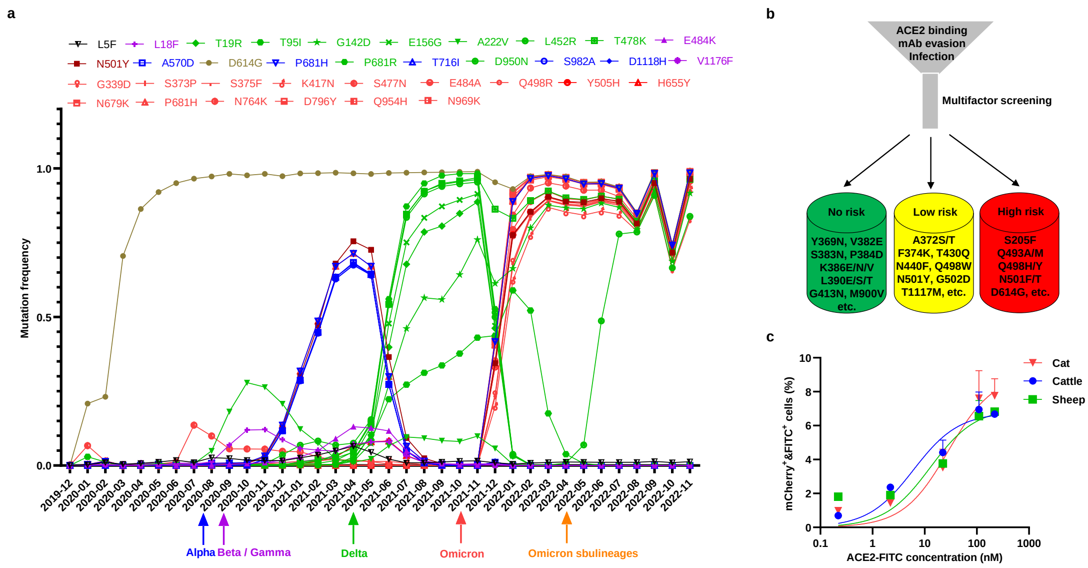

Supplementary Fig. S14:
Keep viral surveillance and continually discover the harmful mutation.

a, The mutation frequency of the top-ranked sites in the real world. The time frame spanned from 2019-12 to 2022-11. About 14 million analysed genomic sequences were downloaded from the GISAID database. The mutation sites that appeared in Alpha, Beta, Gamma, Delta, Omicron, and Omicron sublineages were colored in blue, purple, green, red, and orange, respectively. **b,** The sketch map of multifactor screening in this work, taking ACE2 binding, mAb evasion, and pseudovirus infection into account. The green, yellow, and red battery represented no risk (greatly lost the infectivity versus WT), low risk (comparable or slightly lower infectivity relative to WT), and high risk (higher infectivity than WT), respectively. **c,** The dose-dependent curves of S variants library incubating with three animals' ACE2 (cat, cattle, and sheep) that pre-conjugated with FITC. The percentage of double-positive (mcherry^{+} & FITC^{+}) cells were increased with the adding of ACE2-FITC, so that the S variants with higher adherence could presumably be collected.

Protein sequence

>SARS-CoV-2 WT-S-T2A-mCherry (1273aa WT-S is underlined, of which RRAR and KV are red colored, C terminal 19aa is green highlighted; T2A linker is purple highlighted; mCherry is blue highlighted)

MFVFLVLLPLVSSQCVNLTTRTQLPPAYTNSFTRGVYYPDKVFRSSVLHSTQDLFLPFFSNVTWFHAIHV
SGTNGTKRFDNPVLPFNDGVYFASTEKSNIIRGWIFGTTLDSKTQSLLIVNNATNVVIKVCEFQFCNDPFL
GVYYHKNNKSWMESEFRVYSSANNCTFEYVSQPFLMDLEGKQGNFKNLREFVFKNIDGYFKIYSKHTPI
NLVRDLPQGFSALEPLVDLPIGINITRFQTLLALHRSYLTPGDSSSGWTAGAAAYYVGYLQPRTFLLKYNE
NGTITDAVDCALDPLSETKCTLKSFTVEKGIYQTSNFRVQPTESIVRFPNITNLCPFGEVFNATRFASVYA
WNRKRISNCVADYSVLYNSASFSTFKCYGVSPTKLNDLCFTNVYADSFVIRGDEVRQIAPGQTGKIADYN
YKLPDDFTGCVIAWNSNNLDSKVGGNYNYLYRLFRKSNLKPFERDISTEIYQAGSTPCNGVEGFNCYFP
LQSYGFQPTNGVGYQPYRVVVLSFELLHAPATVCGPKKSTNLVKNKCVNFNFNGLTGTGVLTESNKKFL
PFQQFGRDIADTTDAVRDPQTLEILDITPCSFGGVSVITPGTNTSNQVAVLYQDVNCTEVPVAIHADQLTP
TWRVYSTGSNVFQTRAGCLIGAEHVNNSYECDIPIGAGICASYQTQTNSPRRARSVASQSIIAYTMSLGA
ENSVAYSNNSIAIPTNFTISVTTEILPVSMTKTSVDCTMYICGDSTECSNLLLQYGSFCTQLNRALTGIAVE
QDKNTQEVFAQVKQIYKTPPIKDFGGFNFSQILPDPSKPSKRSFIEDLLFNKVTLADAGFIKQYGDCLGDI
AARDLICAQKFNGLTVLPPLLTDEMIAQYTSALLAGTITSGWTFGAGAALQIPFAMQMAYRFNGIGVTQN
VLYENQKLIANQFNSAIGKIQDSLSSTASALGKLQDVVNQNAQALNTLVKQLSSNFGAISSVLNDILSRLD
KVEAEVQIDRLITGRLQSLQTYVTQQLIRAAEIRASANLAATKMSECVLGQSKRVDFCGKGYHLMSFPQS
APHGVVFLHVTYVPAQEKNFTTAPAICHDGKAHFPREGVFVSNGTHWFVTQRNFYEPQIITTDNTFVSG
NCDVVIGIVNNTVYDPLQPELDSFKEELDKYFKNHTSPDVDLGDISGINASVVNIQKEIDRLNEVAKNLNE
SLIDLQELGKYEQYIKWPWYIWLGFIAGLIAIVMVTIMLCCMTSCCSCLKGCCSCGSCCKFDEDDSEPVL
KGVKLHYTEFTREGRGSLLTCGDVEENPGPDMLMAIIKEFMRFKVHMEGSVNGHEFEIEGEGEGRPYE
GTQTAKLKVTKGGPLPFAWDILSPQFMYGSKAYVKHPADIPDYLKLSFPEGFKWERVMNFEDGGVVTVT
QDSSLQDGEFIYKVKLRGTNFPSDGPVMQKKTMGWEASSERMYPEDGALKGEIKQRLKLKDGGHYDA
EVKTTYKAKKPVQLPGAYNVNIKLDITSHNEDYTIVEQYERAEGRHSTGGMDELYK*

>SARS-CoV-2 tPA-RBD-his (the 5’ terminal tPA signal peptide is green highlighted, the middle 223aa-length RBD is underlined, and the 3’ terminal hexa-histidine tag is blue highlighted)

MDAMKRGLCCVLLLCGAVFVSPSRVQPTESIVRFPNITNLCPFGEVFNATRFASVYAWNRKRISNCVAD
YSVLYNSASFSTFKCYGVSPTKLNDLCFTNVYADSFVIRGDEVRQIAPGQTGKIADYNYKLPDDFTGCVI
AWNSNNLDSKVGGNYNYLYRLFRKSNLKPFERDISTEIYQAGSTPCNGVEGFNCYFPLQSYGFQPTNG
VGYQPYRVVVLSFELLHAPATVCGPKKSTNLVKNKCVNFHHHHHH*

>ACE2-hFC (the extracellular domain of ACE2 is underlined, linker is purple highlighted, hFc is blue highlighted)

MSSSSWLLLSLVAVTAAQSTIEEQAKTFLDKFNHEAEDLFYQSSLASWNYNTNITEENVQNMNNAGDKW
SAFLKEQSTLAQMYPLQEIQNLTVKLQLQALQQNGSSVLSEDKSKRLNTILNTMSTIYSTGKVCNPDNPQ
ECLLLEPGLNEIMANSLDYNERLWAWESWRSEVGKQLRPLYEEYVVLKNEMARANHYEDYGDYWRGD
YEVNGVDGYDYSRGQLIEDVEHTFEEIKPLYEHLHAYVRAKLMNAYPSYISPIGCLPAHLLGDMWGRFW
TNLYSLTVPFGQKPNIDVTDAMVDQAWDAQRIFKEAEKFFVSVGLPNMTQGFWENSMLTDPGNVQKAV
CHPTAWDLGKGDFRILMCTKVTMDDFLTAHHEMGHIQYDMAYAAQPFLLRNGANEGFHEAVGEIMSLSA
ATPKHLKSIGLLSPDFQEDNETEINFLLKQALTIVGTLPFTYMLEKWRWMVFKGEIPKDQWMKKWWEMK
REIVGVVEPVPHDETYCDPASLFHVSNDYSFIRYYTRTLYQFQFQEALCQAAKHEGPLHKCDISNSTEAG
QKLFNMLRLGKSEPWTLALENVVGAKNMNVRPLLNYFEPLFTWLKDQNKNSFVGWSTDWSPYADQSI
KVRISLKSALGDKAYEWNDNEMYLFRSSVAYAMRQYFLKVKNQMILFGEEDVRVANLKPRISFNFFVTAP
KNVSDIIPRTEVEKAIRMSRSRINDAFRLNDNSLEFLGIQPTLGPPNQPPVSPGGDKTHTCPPCPAPELL
GGPSVFLFPPKPKDTLMISRTPEVTCVVVDVSHEDPEVKFNWYVDGVEVHNAKTKPREEQYNSTYRVV
SVLTVLHQDWLNGKEYKCKVSNKALPAPIEKTISKAKGQPREPQVYTLPPSRDELTKNQVSLTCLVKGFY
PSDIAVEWESNGQPENNYKTTPPVLDSDGSFFLYSKLTVDKSRWQQGNVFSCSVMHEALHNHYTQKSL
SLSPGK*

The bioinformatic analysis of the third-generation DNA sequencing data

Annoation：
In the following code, L0 is the name of the sample and can be changed by user. The ref.fa file was a 3762bp-length nucleotide sequence (in this case, Δfurin-S).

Steps:

(1) Extract reads from the bam file to generate the corresponding fastq file:

```
samtools view "./L0.subreads.bam"|perl -ne'chomp;@ar=split(/\t/,$_);{print"\@$ar[0]\n$ar[9]\n+\n$ar[10]\n"}' > ./L0.fastq
```

(2) Prepare the ref.fa file and name it Chromosome P according to the reference genome format. In this study, minimap2 software was used to compare and map the fastq file extracted from the previous step (L0.fastq in this case), and the resulting sam file was obtained (L0.sam in this case). The -a parameter specified the output format, and the -x parameter selected the comparison model.

```
minimap2 -ax map-pb ./ref.fa ./L0.fastq > ./L0.sam
```

(3) The main function of script1 is to extract qualified nucleotide sequences from the sam file (L0.sam in this case) and output it in fasta format. The sequencing reads with too short length or too many bases deleting was filtered out. The last parameter of the script1 was del filtering threshold (30bp in this case), which could be changed by user.

```
perl script1.pl ./L0.sam ./ref.fa ./L0.filter.fa 30
```

(4) The main function of script2 is to convert qualified nucleotide sequences into corresponding amino acid sequences, count the number and print them out. The input is the fasta file filtered in the previous step (L0.filter.fa 30 in this case), and the output is L0.result

```
perl script2.pl ./L0.filter.fa ./L0.result
```

(5) The main function of script3 is to conduct analysis on the obtained amino acid sequence (not considering the sequence with premature stop mutation). The final statistical information included the number of mutations in each sequencing read, the number of sequencing reads for each mutational type, and the number of times each original residue position of the sequence was mutated into other amino acids.

```
perl script3.pl ./L0.result ./L0.stat
```

Supplementary Table S1. Codon usage (*Homo sapiens*)

The codon of 20 natural amino acids (the optimal codon is red highlighted)																			
A	R	N	D	C	E	Q	G	H	I	L	K	M	F	P	S	T	W	Y	V
GCC (0.40)	AGA (0.21)	AAC (0.53)	GAC (0.54)	TGC (0.54)	GAG (0.58)	CAG (0.73)	GGC (0.34)	CAC (0.58)	ATC (0.47)	CTG (0.40)	AAG (0.57)	ATG (1.00)	TTC (0.54)	CCC (0.32)	AGC (0.24)	ACC (0.36)	TGG (1.00)	TAC (0.56)	GTG (0.46)
GCT (0.27)	AGG (0.21)	AAT (0.47)	GAT (0.46)	TGT (0.46)	GAA (0.42)	CAA (0.27)	GGA (0.25)	CAT (0.42)	ATT (0.36)	CTC (0.20)	AAA (0.43)		TTT (0.46)	CCT (0.29)	TCC (0.22)	ACA (0.28)		TAT (0.44)	GTC (0.24)
GCA (0.23)	CGG (0.20)						GGG (0.25)		ATA (0.17)	TTG (0.13)				CCA (0.28)	TCT (0.19)	ACT (0.25)			GTT (0.18)
GCG (0.11)	CGC (0.18)						GGT (0.16)			CTT (0.13)				CCG (0.11)	TCA (0.15)	ACG (0.11)			GTA (0.12)
	CGA (0.11)									TTA (0.08)					AGT (0.15)				
	CGT (0.08)									CTA (0.07)					TCG (0.05)				

A：Alanine
R：Arginine
N：Asparagine
D：Aspartic acid
C: Cystine
E：Glutamic acid
Q：Glutamine
G：Glycine
H：Histidine
I： Isoleucine
L： Leucine
K: Lysine
M：Methionine
F： Phenylalanine
P： Proline
S： Serine
T： Threonine
W：Tryptophan
Y： Tyrosine
V： Valine

Supplementary Table S2. Primers used in this study

Primer name	Sequence (5’-3’) (underlined letter indicated the restriction endonuclease recognition sites, highlighted letter represented the homologous arms for Gibson Assembly)	Application
M13F	TGTAAAACGACGGCCAGT	Amplify ~100nt chip-synthesis oligo pools
M13F	CAGGAAACAGCTATGACC	
pLV-S-F	ATATTAAGGGTTCCAAGCTTAAGCGGCCGCGCCACCATGTTCGTGTTTCTGGTGCTGCTG	Amplify 1254aa-length S variants to pWSLV03
pLV-S-R	AGACTTCCTCTGCCCTCACGCGTGAATTCACAGCAGCTTCCACAAGAACAGCAGC	
pLV-GSAS-F	CAGACAAACTCCCCAggcagcgcctccTCTGTGGCAAGCCAGTCCATCATCG	Mutate RRAR to GSAS (lower case letter)
pLV-GSAS-R	CTGGCTTGCCACAGAggaggcgctgccTGGGGAGTTTGTCTGGGTCTGGTAG	
pLV-PP-F	CTGAGCCGGCTGGACcctcccGAGGCAGAGGTGCAGATCGACCGGC	Mutate KV to PP (lower case letter)
pLV-PP-R	CTGCACCTCTGCCTCgggaggGTCCAGCCGGCTCAGGATATCATTC	
pCD-S-F	ACTTAAGCTTGGTACCGAGCTCGGATCCGCCACCATGTTCGTGTTTCTGGTGCTGCTG	Amplify 1273aa-length S variants to pCDNA3.1(+)
pCD-S-R	GGGTTTAAACGGGCCCTCTAGACTCGAGTTAGGTGTAGTGCAGCTTCACGCCCTTC	
pCD-RBD-1F	tgctgtgtgctgctgctgtgtggagcagtcttcgtttcgcccagcAGGGTGCAGCCTACCGAGTCCATCG	Amplify 223aa-length RBD variants to pCDNA3.1(+) with 5’ tPA signal peptide (lower case letter) and 3’ hexa-histidine tag (lower case italic letter)
pCD-RBD-2F	ACTTAAGCTTGGTACCGAGCTCGGATCCGCCACCATGgatgcaatgaagagagggctctgctgtgtgctgctgctgtgtggag	
pCD-RBD-R	GGGTTTAAACGGGCCCTCTAGACTCGAGTTA*atggtgatggtgatggtg*GAAGTTCACGCACTTGTTCTTCACC	
S-seq-1F	CTGCCCATCGGCATCAACATCAC	Verify the correct mutation of each S variants
S-seq-2F	TTCCAGCCAACCAACGGCGTGG	
S-seq-3F	GAATAGAGCCCTGACAGGCATCG	
S-seq-4F	CTGGGCCAGTCCAAGAGAGTGG	
S-seq-1R	GCTGCAGATAGCCCACATAGTAG	
RBD-seq-F	CTCTGGCTAACTAGAGAACCCACTG	Verify the correct mutation of each RBD variants
RBD-seq-R	ACCTTCCAGGGTCAAGGAAGGCACG	
RT-GSP	TCGAACTCGTGGCCGTTCAC	Reverse transcription of total RNA sample
cDNA-F	GTTATATTAAGGGTTCCAAGCTTAAGC	Amplify the first strand of cDNA
cDNA-R	CCACGTCACCGCATGTTAGTAGAC	
qPCR-GAPDH-F	TGCACCACCAACTGCTTAGC	Quantify the expression of GAPDH
qPCR-GAPDH-R	GGCATGGACTGTGGTCATGAG	
qPCR-S-F	ATCTTTGGCACCACACTGGAC	Quantify the expression of S variants
qPCR-S-R	AACTCGCTCTCCATCCAAGACT	
qPCR-VSV-F	TCTCGTCTGGATCAGGCGG	Quantify the titer of S variants pseudovirus
qPCR-VSV-R	TGCTCTTCCACTCCATCCTCTTGG	

Supplementary Table S3. The third-generation sequencing reads

Experiment	The collected mCherry$^+$ cells for screening (MOI ~0.1)	Totally obtained CCS reads (Accuracy >99%)			Finally analysed CCS reads (Unique reads are underlined)			Average mutation per read		
		S_{I}	S_{II}	S_{III}	S_{I}	S_{II}	S_{III}	S_{I}	S_{II}	S_{III}
Rep. 1	7.5 X 10^6	3.87 M	3.76 M	0.61 M	2.96 M (1.64 M)	3.06 M (0.98 M)	0.49 M (0.20 M)	2.35 aa	1.77 aa	2.34 aa
Rep. 2	1.0 X 10^7	3.62 M	3.82 M	0.81 M	3.00 M (1.66 M)	3.29 M (1.08 M)	0.67 M (0.29 M)	2.32 aa	1.88 aa	2.53 aa

Supplementary Table S4. The relevant work on the screening of RBD, S variants, or its derived mAbs

**Species**	**Technical route**	**Application**	**Mutational range**	**Throughput**	**Analysis strategy**	**Vital mutational types**	**Ref.**
Yeast (Saccharomyces cerevisiae)	Incubate with dimer ACE2; FACS screen; Titration curves verify	Reveal the feature of RBD folding and ACE2 binding	RBD (331-531aa) Mutate 201aa	~ 10^5 barcoded variants	Link each RBD variant to its barcode via long-read PacBio SMRT sequencing; then deep sequencing of variant barcodes	Low affinity (L455Y, Y449F, N501D, G502D, etc.) High affinity (N501F, N501T, Q498Y, Q498H etc.)	[28]
Yeast (Saccharomyces cerevisiae)	Incubate with ACE2; FACS screen; Titration curves verify	S-variants prediction; Antiviral drug design	RBD (336-528aa) RBD (431-528aa) RBD (336-528aa) 3 cycle mutating	~ 10^4 variants	Sanger sequencing	RBD62 drug (I358F, V445K, N460K, I468T, T470M, S477N, E484K, Q498R, N501Y) 1000-fold higher affinity	[29]
Yeast (Saccharomyces cerevisiae)	Incubate with 10 mAbs; FACS screen	Map antibody-escape mutation	RBD (331-531aa) Mutate 201aa	~ 10^5 barcoded variants	Link each RBD variant to its barcode via long-read PacBio SMRT sequencing; then deep sequencing of variant barcodes	C361, K378, V382, S383, P384, F392, D420, A475, E484, F486, N487, F490, G496, Q498, T500, etc.	[30]
Yeast (Saccharomyces cerevisiae)	Incubate with 5 mAbs; FACS screen	Identify antibody-escape mutants	RBD (333-537aa) Mutate 119aa that were surface-exposed	~2000 mutations	Isolate genomic DNA, amplify the certain region, send for the next-generation sequencing	Escape positions K417, D420, Y421, F486, and Q493 as notable hotspots	[31]
Yeast (Saccharomyces cerevisiae)	Incubate with 7 mAbs; FACS screen	Map antibody-escape mutation by different classes of mAbs	RBD (331-531aa) Mutate 201aa	~ 10^5 barcoded variants	Link each RBD variant to its barcode via long-read PacBio SMRT sequencing; then deep sequencing of variant barcodes	R346, K417, K444, Y449, L452, L455, F456, N460, A475, E484, N486, F490, etc.	[32]

Mice (Genetically humanized VelocImmune (VI) breed)	Immunized with a DNA plasmid that expressed S and boosted with a recombinant protein that was composed of RBD; FACS screen	Yield a SARS-CoV-2 antibody cocktail	Spleens from mice were subjected to biotin-labeled monomeric RBD antigen	Naturally paired heavy and light chain cDNAs were cloned, then transfect CHO cells to produce >200 antibody	The antibody variable regions were sequenced by next-generation sequencing, and the repertoire for heavy and light chain pairs was identified; The selected four antibodies displayed ~37.1 to 42.8pM affinity against trimeric S	VH3-53 paired with VKI-9, VKI-33, or VKI-39	[57]
Human (B cells)	Survivor that has been infected by SARS-CoV-2; FACS screen		Whole blood was collected from patients; Sort RBD-specific B cells			VH3-66 paired with VKI-33 VH3-70 paired with VKI-39	
Yeast (Saccharomyces cerevisiae)	Incubate with monomer ACE2; FACS screen	Understand how epistasis shifts the effects of mutations	RBD (331-531aa) WT/Alpha/Beta/Delta/Eta backbone	~10^5 barcoded variants	Link each RBD variant to its barcode via long-read PacBio SMRT sequencing; then deep sequencing of variant barcodes	Q498R, N501Y, G446, Y449	[62]
Mammalian (HEK293T)	Spike display; Flow cytometry	Evaluate the evasion of various mAbs	Ectodomain (1-1208aa), but only consider the clinically circulating NTD variants	~200 variants	Test one S variant at a time	Y145, K147, and W152 are public epitopes of 4-A8, CM17, CM25, and 1-6	[64]
Mammalian (HEK293T)	Fluorescence-based sorting	S-based immunogen design (with increased expression)	Based on S-2P Mutate 14-301aa Cover NTD domain	Thousands of single mutations in parallel	Isolate genomic DNA, amplify the certain region, send for the next-generation sequencing	S50Q and G232E	[65]

Mammalian (HEK293T)	Fluorescence-based fusion assay;<br>To detect the green-fluorescent syncytia	Vaccine design (prefusion-stabilized)<br>High expression but Low fusogenicity	Based on S-2P;<br>Mutate 883-1034aa;<br>Cover HR1 (912-984aa) and CH (985-1034aa)	Thousands of single mutations in parallel	Isolate genomic DNA, amplify the certain region, send for the next-generation sequencing	A892P, A942P, and D994E/Q	[66]
Mammalian (HEK293T-rtTA)	Non-replicative VSV-G pseudotyped lentivirus	Identify mutation that impact the antibody neutralization and pseudovirus infection	Full-length S scale, but only consider the mutations that occurred in GISAID	~135000 unique mutation combinations	First perform long-read PacBio sequencing to link the barcodes to the full set of spike mutations for each variant; then conduct short-read Illumina sequencing of the barcode in all subsequent experiments	N439, K444, G446, G447, P499 for mAb LYCoV1404;<br>S172, L176, D178 for mAb 5-7;<br>D1146, D1153, F1156 for mAb CC9.104, and CC67.105	[67]
Mammalian (HEK293T)	Fluorescence-based sorting (mCherry & FITC); Incubate with dimer ACE2 and mAb REGN10987	Discover the key residues of tighter ACE2 affinity and higher mAb evasion	Based on Δfurin-S;<br>Full-length S scale;<br>Mutate 1-1254aa	10^6~10^7 combinations of mutations/ cycle	Isolate the total RNA, reverse-transcribed with GSP, amplification with low cycles, and send for the third-generation DNA sequencing	S205F, Y453F, Q493A, Q493M, Q498H, Q498Y, N501F, N501T, etc. mutational types that enhance infectivity;<br>Y453, Q493, Q498, etc. residues that escape antibody	This work
